# Relationship Between a Plant‐Based Dietary Portfolio and Risk of Cardiovascular Disease: Findings From the Women's Health Initiative Prospective Cohort Study

**DOI:** 10.1161/JAHA.121.021515

**Published:** 2021-08-04

**Authors:** Andrea J. Glenn, Kenneth Lo, David J. A. Jenkins, Beatrice A. Boucher, Anthony J. Hanley, Cyril W. C. Kendall, JoAnn E. Manson, Mara Z. Vitolins, Linda G. Snetselaar, Simin Liu, John L. Sievenpiper

**Affiliations:** ^1^ Department of Nutritional Sciences University of Toronto Ontario Canada; ^2^ Clinical Nutrition and Risk Factor Modification Center St. Michael's Hospital Toronto Ontario Canada; ^3^ Toronto 3D Knowledge Synthesis and Clinical Trials Unit St. Michael's Hospital Toronto Ontario Canada; ^4^ Department of Applied Biology and Chemical Technology The Hong Kong Polytechnic University Hung Hom Hong Kong China; ^5^ Centre for Global Cardiometabolic Health Brown University Providence RI; ^6^ Li Ka Shing Knowledge Institute St. Michael's Hospital Toronto Ontario Canada; ^7^ Division of Endocrinology and Metabolism St. Michael's Hospital Toronto Ontario Canada; ^8^ Dalla Lana School of Public Health and Department of Medicine University of Toronto Ontario Canada; ^9^ Leadership Sinai Centre for Diabetes Mount Sinai Hospital Toronto Ontario Canada; ^10^ College of Pharmacy and Nutrition University of Saskatchewan Saskatoon Saskatchewan Canada; ^11^ Channing Division of Network Medicine Department of Medicine Brigham and Women's Hospital and Harvard Medical School Boston MA; ^12^ Department of Epidemiology Harvard T.H. Chan School of Public Health Boston MA; ^13^ Division of Preventive Medicine Brigham and Women's Hospital and Harvard Medical School Boston MA; ^14^ Department of Epidemiology and Prevention Wake Forest School of Medicine Winston‐Salem NC; ^15^ Department of Epidemiology University of Iowa College of Public Health Iowa City IA; ^16^ Division of Endocrinology Department of Medicine, and Division of Cardiothoracic Surgery Department of Surgery The Warren Alpert School of Medicine and Rhode Island Hospital Providence RI; ^17^ Department of Epidemiology Brown University School of Public Health Providence RI

**Keywords:** cardiovascular disease, dietary patterns, dietary portfolio, plant‐based, prospective cohort study, Cardiovascular Disease, Diet and Nutrition, Epidemiology, Primary Prevention

## Abstract

**Background:**

The plant‐based Dietary Portfolio combines established cholesterol‐lowering foods (plant protein, nuts, viscous fiber, and phytosterols), plus monounsaturated fat, and has been shown to improve low‐density lipoprotein cholesterol and other cardiovascular disease (CVD) risk factors. No studies have evaluated the relation of the Dietary Portfolio with incident CVD events.

**Methods and Results:**

We followed 123 330 postmenopausal women initially free of CVD in the Women's Health Initiative from 1993 through 2017. We used Cox proportional‐hazard models to estimate adjusted hazard ratios (HRs) and 95% CI of the association of adherence to a Portfolio Diet score with CVD outcomes. Primary outcomes were total CVD, coronary heart disease, and stroke. Secondary outcomes were heart failure and atrial fibrillation. Over a mean follow‐up of 15.3 years, 13 365 total CVD, 5640 coronary heart disease, 4440 strokes, 1907 heart failure, and 929 atrial fibrillation events occurred. After multiple adjustments, adherence to the Portfolio Diet score was associated with lower risk of total CVD (HR, 0.89; 95% CI, 0.83–0.94), coronary heart disease (HR, 0.86; 95% CI, 0.78–0.95), and heart failure (HR, 0.83; 95% CI, 0.71–0.99), comparing the highest to lowest quartile of adherence. There was no association with stroke (HR, 0.97; 95% CI, 0.87–1.08) or atrial fibrillation (HR, 1.10; 95% CI, 0.87–1.38). These results remained statistically significant after several sensitivity analyses.

**Conclusions:**

In this prospective cohort of postmenopausal women in the United States, higher adherence to the Portfolio Diet was associated with a reduction in incident cardiovascular and coronary events, as well as heart failure. These findings warrant further investigation in other populations.

**Nonstandard Abbreviations and Acronyms**
FFQ: food frequency questionnaire
MUFAs: monounsaturated fatty acids
OS: observational study
WHI: Women's Health Initiative

**Clinical Perspective**

**What Is New?**

- Higher adherence to the Portfolio Diet was associated with a 11%, 14%, and 17% lower risk of total cardiovascular disease, coronary heart disease, and heart failure, respectively, but no association was seen with stroke or atrial fibrillation.
- This study shows that the beneficial effects of the Portfolio Diet on cardiovascular risk factors from the clinical trials may translate into lower hard clinical cardiovascular disease events.

**What Are the Clinical Implications?**

- Given the increased interest in plant‐based foods and diets around the world, and growing concerns related to ethical and environmental implications of diet, the Portfolio Diet warrants attention from healthcare professionals as another therapeutic dietary approach for cardiovascular disease risk reduction.

The Dietary Portfolio, or Portfolio Diet, is a plant‐based dietary pattern that was developed in the early 2000s to lower low‐density lipoprotein cholesterol (LDL‐C).^1^, ^2^, ^3^, ^4^, ^5^, ^6^ The underlying diet is low in saturated fat and cholesterol (National Cholesterol Education Program Step II diet^7^), with the addition of a “portfolio” of 4 cholesterol‐lowering foods and nutrients: nuts, plant protein (soy and pulses), viscous fiber (oats, barley, psyllium, eggplant, okra, apples, oranges, and berries), and phytosterols (originally provided as enriched margarine). An extension of the diet includes adding monounsaturated fats (MUFAs; such as olive/canola oil or avocado).^6^ Early findings from a metabolically controlled randomized trial showed that the LDL‐C lowering effect of the Portfolio Diet was similar to the control diet taken with 20mg lovastatin (−28.6% versus −30.9%).^3^ Recently, a systematic review and meta‐analysis of metabolically controlled and ad libitum trials showed that the Portfolio Diet significantly lowered LDL‐C by 17% (27% in the intended combination with an National Cholesterol Education Program Step II diet). It also lowered other cardiovascular disease (CVD) risk factors, including the alternate blood lipid targets of non‐high‐density lipoprotein cholesterol by 14% and ApoB (apolipoprotein B) by 15%, and CRP (C‐reactive protein) by 32%.^8^ These benefits have been recognized by CVD and diabetes mellitus clinical practice guidelines internationally, including those of the Canadian Cardiovascular Society,^9^ Diabetes Canada,^10^ European Atherosclerosis Society,^11^ and Heart UK.^12^

Currently, it is not known if these beneficial effects of the diet translate into lower risk of clinical CVD events. The individual components of the Portfolio Diet have been found to be associated with lower incidence of CVD events in prospective cohorts,^13^, ^14^, ^15^, ^16^, ^17^ and 2 components of the diet (nuts and extra virgin olive oil) were shown to reduce major vascular events in the landmark PREDIMED (Prevención con Dieta Mediterránea) trial compared to a low saturated fat^18^ however, the additive/combined effects of the Portfolio Diet components have not been assessed with incident CVD. Although conducting a long‐term randomized trial with CVD as the primary outcome would be preferable, this type of trial is not yet feasible. Analyses of established observational studies may be helpful in assessing the long‐term effectiveness of the Portfolio Diet. We have therefore developed a scoring system to measure adherence to the Portfolio Diet for use in these study designs. Here, for the first time, we have evaluated the association of a Portfolio Diet score with CVD outcomes in the WHI (Women's Health Initiative).

## METHODS

### Study Population and Design

**Figure 1:**
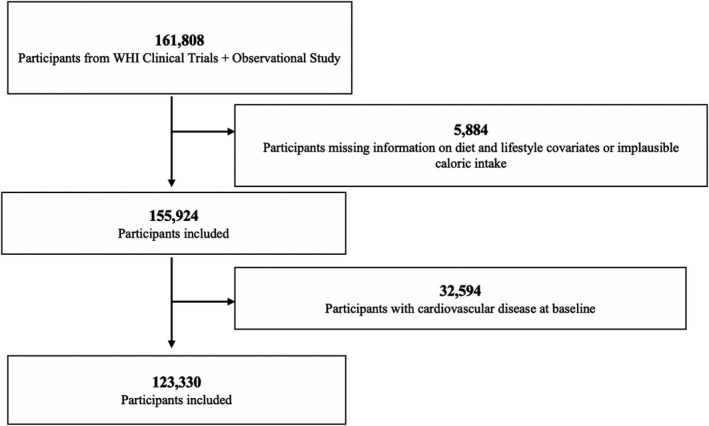
Flow chart for study sample, WHI (Women's Health Initiative) cohort, 1993 to 2017.

The design and methods of the WHI have been published elsewhere.^19^, ^20^, ^21^ Briefly, between 1993 and 1998, postmenopausal women aged 50 to 79 years were recruited into clinical trials or an observational study (OS) (n=161 808). Recruitment and baseline data collection have been previously reported.^20^ This analysis includes follow‐up through February 28, 2017. We excluded participants who had a history of CVD at baseline (n=32 594), and missing information regarding diet and lifestyle covariates or implausible caloric intake (<600 kcal or >5000 kcal/day) (n=5884). The final analysis included 123 330 women (Figure 1). The baseline characteristics of participants included or excluded due to missing data from the analysis are shown in Table S1. Written informed consent was obtained from all WHI participants and procedures were approved by institutional review boards at all participating institutions. The WHI data are accessible to qualified researchers trained in human subject confidentiality protocols and requests to access the data set may be sent to the WHI Publications and Presentations Committee.

### Dietary Assessment

The exposure was diet as measured by a Portfolio Diet score. The foods and nutrients composing this score were self‐reported using the food frequency questionnaire (FFQ) developed and validated for the WHI^22^, ^23^ at enrollment and again at year 3 for the OS participants. No further diet assessments were available for the WHI participants. We used a cumulative average score for those who completed the FFQ at baseline and year 3 (Data S1).

Food items on the WHI FFQ that are characteristic of the Portfolio Diet were categorized into 6 components (plant protein, nuts, viscous fiber, phytosterols, MUFAs, and saturated fat/cholesterol sources). Intake was assessed as servings/day of targeted foods in all components except phytosterols, which used all FFQ food items to derive total daily intake (mg/day). For the 6 components, each was scored from 1 (unhealthy) to 5 (healthiest) according to participant's quintile of intake resulting in a score range between 6 and 30, with higher scores indicating higher adherence to the Portfolio Diet. Additional information on the Portfolio Diet score development is provided in Data S1 and Table S2.

### Ascertainment of CVD Outcomes

Our primary outcomes included total CVD, coronary heart disease (CHD; defined as clinical myocardial infarction, definite silent myocardial infarction, or a death due to definite CHD or possible CHD), and stroke incidence and death as these CVDs are causally related to high LDL‐C and the Portfolio Diet has an established cholesterol‐lowering effect.^8^ Total CVD was a composite of nonfatal myocardial infarction, CHD death, stroke, coronary revascularization and incident heart failure (HF).^24^ Our secondary, or exploratory, outcomes included HF and atrial fibrillation (AF). The outcomes were ascertained in the WHI through self‐reported medical questionnaires completed by participants every 6 to 12 months, depending on study assignment. Medical records and death certificates for all outcomes were reviewed by central physician adjudicators or trained local adjudicators.^25^

### Covariates

Covariates that were included in our models were based on information on the participants' lifestyle and risk factors for CVD assessed at baseline, including age, region in the United States, race/ethnicity, alcohol intake, physical activity, caloric intake, sodium intake, hysterectomy history, body mass index (BMI), hormone therapy use, personal history of hypertension and high cholesterol, family history of CVD and diabetes mellitus, diabetes mellitus or cancer diagnoses, smoking status, education, marital status, and clinical trial/study arm. Detailed descriptions of the validity and reproducibility of baseline measurements have been previously published.^21^

### Statistical Analysis

Baseline characteristics were described by quartile of the Portfolio Diet score using means with SDs for continuous variables and frequencies with percentages for categorical variables. To compare baseline characteristics, χ^2^ tests were used for categorical variables and analysis of variance for continuous variables.

Participants were categorized into quartiles of the Portfolio Diet score, with the lowest quartile serving as the reference group, as per our prespecified analysis plans. Cox proportional hazard models were used to estimate hazard ratios (HRs) and 95% CIs for the association between the Portfolio Diet score quartiles and CVD outcomes. Two multivariable models were used. Covariates commonly examined in studies of dietary pattern scores and CVD risk were included based on our a priori analysis plan. Model 1 was adjusted for age (continuous), region (Northeast, South, Midwest, West), smoking (never, past, current), and study arm (hormone replacement therapy arm, dietary modification arm, calcium and vitamin D) arm). Model 2 was adjusted for model 1+race/ethnicity (White, Black, Hispanic, Asian/Pacific Islander, Other [American Indian, Alaskan Native, other]), education (college or above, below college), marital status (presently married/other), hysterectomy history (yes/no), body mass index (continuous), physical activity (continuous), alcohol intake (>7 drinks/week, <7 drinks/week), energy intake (continuous), cancer status (yes/no), hypertension status (yes/no), diabetes mellitus status (yes/no), sodium intake (continuous), family history of CVD (yes/no), family history of diabetes mellitus (yes/no), postmenopausal hormone use (never, past, current), and cholesterol‐lowering medication use (yes/no). For all covariates, 5% or less of values were missing. When we checked the proportional hazard model assumptions using Schoenfeld residuals method, no violations of the assumption were found.

Tests for linear trend were conducted by assigning the median value to each quartile. Our main analysis (per our protocol) included all WHI participants (clinical trials+OS). We also conducted several sensitivity analyses to test the robustness of our main findings. First, we conducted analyses by restricting the data to the OS participants only as the clinical trials participants have received an intervention and may be different from the OS participants. We also then (1) restricted analyses to the baseline diet only, (2) excluded participants from the dietary modification trial (a low fat diet intervention), as their diet may have changed overtime, (3) excluded CVD events within the first 3 years of follow‐up to address possible reverse causation, (4) excluded those with diabetes mellitus at baseline due to their higher CVD risk, and (5) completed multiple imputation for missing covariate data (using the multivariate imputation by chained equations method).^26^ We also conducted post hoc sensitivity analyses where we created another Portfolio Diet score based on the recommendations from the Portfolio Diet randomized clinical trials (further details included in Table S3). We then also applied subgroup analyses according to several potential interactive factors (age, body mass index, family history of CVD, race/ethnicity, smoking status, and cholesterol‐lowering medication) and conducted interaction tests via multiplicative interaction terms using model 2 to assess if the *P* for interactions were significant. Additional analyses we conducted included evaluating associations between the 6 individual components of the Portfolio Diet and risk of the CVD outcomes. Statistical tests were 2‐sided and *P*<0.05 was considered statistically significant. The statistical analyses were conducted with Stata statistical software (Stata Statistical Software: Release 15., Stata Corp., College Station, TX). Further information on the methods can be found in Data S1.

## RESULTS

### Lifestyle Characteristics of the Participants

Baseline characteristics by quartiles of the Portfolio Diet score are shown in Table 1. Women with higher scores tended to be older, have a lower body mass index, engage in more physical activity, have a higher education, be less likely to smoke, as well as several other differences. All of these known risk factors at baseline were adjusted for in our analyses. Mean intake of the Portfolio Diet score components is shown in Table 2. The included participants were different from the excluded participants (eg, Black or Hispanic, 10 118 [8.2%] and 4875 [4.0%] versus 1061 [19.0%] and 657 [10.2%], respectively; and above college education, 83 887 [68.5%] versus 3086 [55.9%]) (Table S1).

**Table 1:** Baseline Characteristics of 123 330 Participants in the WHI According to Quartiles of the Portfolio Diet Score

Mean (SD)/No. (%)	Q1 (6–14)	Q2 (14.5–17)	Q3 (17.5–20)	Q4 (20.5–30)	*P* Value
Number of participants	32 403	33 713	30 755	26 459	
Time‐to‐event/censored in years	14.9 (5.79)	15.4 (5.67)	15.5 (5.61)	15.6 (5.58)	<0.001
Age, y	62.2 (7.05)	62.6 (7.07)	62.9 (7.17)	63.1 (7.27)	<0.001
Body mass index, kg/m^2^	28.7 (6.08)	28.0 (5.81)	27.6 (5.69)	26.7 (5.54)	<0.001
Recreational physical activity (MET‐h/wk)	9.60 (12.01)	11.99 (13.2)	13.64 (14.15)	16.77 (15.63)	<0.001
Dietary energy, kcal/d	1368 (522)	1577 (603)	1755 (640)	1933 (665)	<0.001
Region in the United States					
Northeast	9635 (29.7)	8066 (23.9)	6331 (20.6)	4459 (16.9)	<0.001
South	8261 (25.5)	8804 (26.1)	7951 (25.9)	6360 (24.0)	
Midwest	7794 (24.1)	8018 (23.8)	6706 (21.8)	4588 (17.3)	
West	6713 (20.7)	8825 (26.2)	9767 (31.8)	11 052 (41.8)	
Race/ethnicity					
White	26 517 (82.0)	28 582 (85.0)	26 019 (84.8)	22 166 (84.0)	<0.001
Black	3869 (12.0)	2757 (8.2)	2106 (6.9)	1368 (5.3)	
Hispanic	1023 (3.2)	1229 (3.7)	1337 (4.4)	1286 (4.9)	
Asian/Pacific Islander	582 (1.8)	714 (2.1)	881 (2.9)	1213 (4.6)	
Alcoholic drinks					
>7 drinks/wk	3789 (11.7)	4217 (12.6)	3782 (12.3)	3314 (12.6)	0.003
Sodium intake, mg/d	2204 (888)	2607 (1044)	2953 (1142)	3329 (1240)	<0.001
Hormone therapy use					
Never	12 055 (38.3)	10 853 (33.2)	9592 (32.3)	7550 (29.5)	<0.001
Past	7404 (23.5)	7270 (22.2)	6334 (21.3)	5586 (21.8)	
Current	12 021 (38.2)	14 602 (44.6)	13 830 (46.5)	12 500 (48.8)	
Hysterectomy ever	13 230 (40.8)	13 607 (40.4)	12 228 (39.8)	10 107 (38.2)	<0.001
Treated high cholesterol	3284 (10.8)	3730 (11.8)	3450 (12.0)	3002 (12.0)	<0.001
History of hypertension	10 396 (32.3)	10 208 (30.5)	8981 (29.4)	7044 (26.8)	<0.001
History of cancer	2707 (8.4)	2829 (8.5)	2680 (8.8)	2296 (8.8)	0.223
Family history diabetes mellitus	10 583 (32.8)	10 685 (31.8)	9626 (31.4)	7770 (29.4)	<0.001
Family history of cardiovascular disease	20 816 (64.2)	21 898 (64.9)	20 248 (65.8)	17 167 (64.9)	<0.001
Self‐reported diabetes mellitus	1578 (4.9)	1603 (4.8)	1442 (4.7)	1118 (4.2)	0.001
Smoking status					
Never	15 706 (48.5)	17 049 (50.6)	16 253 (52.9)	14 323 (54.1)	<0.001
Past	13 281 (50.0)	142 401 (42.2)	12 891 (41.9)	11 166 (42.2)	
Current	3416 (10.5)	2424 (7.20)	1611 (5.2)	970 (3.7)	
Education: college or above	19 165 (59.6)	22 256 (66.5)	21 918 (71.8)	20 548 (78.2)	<0.001
Marital status: present relationship	19 785 (61.3)	21 640 (64.5)	20 032 (654)	16 946 (64.3)	<0.001
Hormone replacement therapy arm					
Not randomized	25 436 (78.6)	27 904 (82.8)	25 845 (84.0)	22 468 (84.9)	<0.001
E‐alone	1368 (4.2)	1055 (3.1)	924 (3.0)	640 (2.4)	
E‐alone control	1442 (4.5)	1076 (3.2)	826 (2.7)	754 (2.9)	
E+P intervention	2083 (6.4)	1930 (5.7)	1653 (5.4)	1306 (4.9)	
E+P control	2074 (6.4)	1748 (5.2)	1507 (4.9)	1291 (4.9)	
Dietary modification arm					
Not randomized	20 972 (64.7)	22 964 (68.1)	21 349 (69.4)	18 958 (71.7)	<0.001
Intervention	4472 (13.8)	4268 (12.7)	3867 (12.6)	2981 (11.3)	
Control	6959 (21.5)	6481 (19.2)	5539 (18.0)	4520 (17.1)	
Calcium and vitamin D arm					
Not randomized	23 646 (73.0)	25 738 (76.3)	23 738 (77.2)	20 778 (78.5)	<0.001
Intervention	4398 (13.6)	3982 (11.8)	3529 (11.5)	2865 (10.8)	
Control	4359 (13.5)	3993 (11.8)	3477 (11.3)	2816 (10.6)	

E+P indicates estrogen plus progestin; E‐alone, estrogen‐alone; Q, quartile; and WHI, Women's Health Initiative

**Table 2:** Scoring Criteria for the Portfolio Diet Score From Targeted Foods in Each Component and Mean* Daily Intake^†^ for Each Quintile

Component	Main Targeted Foods From WHI FFQ^‡^	Scoring Criteria				
		Q1 (1 Point), servings/d	Q2 (2 Points), servings/d	Q3 (3 Points), servings/d	Q4 (4 Points), servings/d	Q5 (5 Points), servings/d
Plant protein	Soy beverage; green peas; refried beans; all other beans; tofu and textured vegetable products; bean soups	0.05	0.13	0.21	0.34	0.77
Viscous fiber	Oranges, grapefruit and tangerines; apples and pears; strawberries; okra; oats	0.14	0.38	0.64	0.98	1.78
Nuts	Peanut butter, peanuts, other nuts and seeds	0.00	0.04	0.10	0.23	0.62
Phytosterols	Estimated from all plant foods	133 mg	191 mg	236 mg	288 mg	404 mg
MUFAs	Olive or canola oil; avocado and guacamole	0.00	…^§^	0.01	0.03	0.25
Saturated fat/cholesterol^‖^	High fat dairy; eggs; chicken/turkey with skin; red and processed meats; organ meats; gravy; butter	4.19	2.04	1.34	0.86	0.38

FFQ indicates food frequency questionnaire; MUFAs, monounsaturated fatty acids; and Q, quintile.

* Mean of baseline and year 3 FFQ, when possible.

^†^ All components reported as servings/day except for phytosterols (mg/day).

^‡^ Full list of FFQ food items in Table S1 in the Supplementary Appendix.

^§^ Two points not given to any participants based on consumption on MUFAs (low in entire population).

^‖^ Higher quintiles represent higher intake; however, high intake and high quintiles of saturated fat/cholesterol received lower scores.

### Portfolio Diet Score and CVD Outcomes

**Figure 2:**
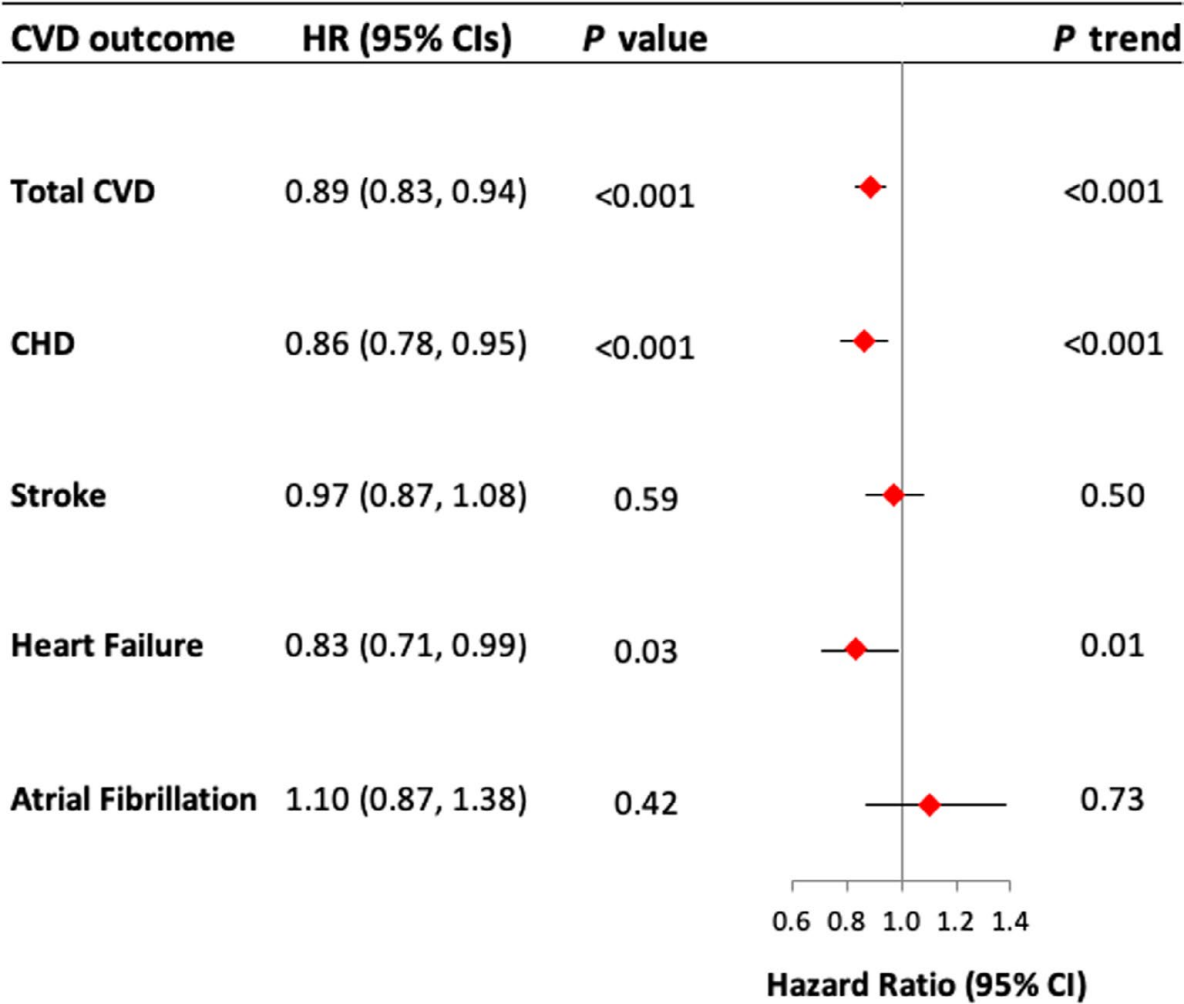
Summary of findings of incident cardiovascular disease, coronary heart disease, stroke, heart failure, and atrial fibrillation comparing low to high adherence to the Portfolio Diet. Hazard ratios (HRs) and for comparing participants in Q1 (low adherence [reference category]) to Q4 (high adherence) of the Portfolio Diet with CVD outcomes in the Women's Health Initiative (Clinical Trials+Observational Study). Multivariate‐adjusted models were adjusted for the following: age, region, smoking, clinical trial study arm, ethnicity, education, marital status, hysterectomy history, body mass index, physical activity, alcohol intake, energy intake, cancer status, hypertension status, diabetes mellitus status, sodium intake, family history of CVD, family history of diabetes mellitus, hormone therapy use, and cholesterol‐lowering medication use. *P* trend was determined by assigning a median value to each quartile. Horizontal lines represent 95% CIs. CHD indicates coronary heart disease; and CVD, cardiovascular disease.

During an average of 15.3 years of follow‐up, we documented 13 365 incident CVD cases, including 5640 CHD cases, 4400 stroke cases, 1907 HF cases, and 929 AF cases. After adjusting for potential confounders, we observed that women in the top quartile (Q4) of the Portfolio Diet score, compared to those in the bottom quartile (Q1), had an HR of 0.89 (95% CI, 0.83–0.94; *P*<0.001 for trend) for risk of total CVD, 0.86 (95% CI, 0.78–0.95; *P*<0.001 for trend) for risk of CHD, and 0.97 (95% CI, 0.87–1.08; *P*=0.50 for trend) for stroke (Table 3 and Figure 2). For our exploratory outcomes, we observed that women in the top quartile compared to those in the bottom quartile had an HR of 0.83 (95% CI, 0.71–0.99; *P*=0.01 for trend) for HF and 1.10 (95% CI, 0.87–1.38; *P*=0.73 for trend) for AF (Table 3 and Figure 2). Absolute incidence rates per 100 000 person‐years among quartiles of adherence are shown in Tables 3 and 4.

**Table 3:** Prospective Association of the Portfolio Diet Score With Risk of Cardiovascular Disease Outcomes Among 123 330 Participants in the Women's Health Initiative (CT+OS) (1993–2017)

	Cases/Total	Person‐Years	Incidence Rate (Per 100 000 Person‐Years)	Model 1* (n=123 330)		Model 2^†^ (n=104, 894)	
				HR (95% CI)	*P* Value	HR (95% CI)	*P* Value
Total CVD							
Q1 (6–14)	3872/32 403	459 280	834	1.00 [reference]		1.00 [reference]	
Q2 (14.5–17)	3758/33 713	493 872	760	0.92 (0.88–0.96)	<0.001	0.97 (0.92–1.02)	0.259
Q3 (17.5–20)	3189/30 755	456 016	699	0.84 (0.80–0.88)	<0.001	0.91 (0.86–0.96)	0.001
Q4 (20.5–30)	2549/26 459	396 421	643	0.77 (0.74–0.82)	<0.001	0.89 (0.83–0.94)	<0.001
*P* trend							<0.001
CHD							
Q1 (6–14)	1697/32 403	474 873	357	1.00 [reference]		1.00 [reference]	
Q2 (14.5–17)	1528/33 713	510 244	299	0.85 (0.80–0.91)	<0.001	0.92 (0.85–0.99)	0.029
Q3 (17.5–20)	1328/30 755	469 623	282	0.80 (0.74–0.86)	<0.001	0.85 (0.78–0.93)	<0.001
Q4 (20.5–30)	1087/26 459	406 937	267	0.75 (0.69–0.81)	<0.001	0.86 (0.78–0.95)	0.002
*P* trend							<0.001
Stroke							
Q1 (6–14)	1192/32 403	476 881	250	1.00 [reference]		1.00 [reference]	
Q2 (14.5–17)	1256/33 713	511 321	246	0.99 (0.91–1.07)	0.811	1.03 (0.95–1.13)	0.449
Q3 (17.5–20)	1061/30 755	471 010	225	0.89 (0.82–0.97)	0.008	0.97 (0.88–1.07)	0.545
Q4 (20.5–30)	892/26 459	407 674	219	0.86 (0.79–0.94)	0.001	0.97 (0.87–1.08)	0.598
*P* trend							0.500
Heart failure							
Q1 (6–14)	567/32 403	479 309	118	1.00 [reference]		1.00 [reference]	
Q2 (14.5–17)	566/33 713	513 986	110	0.96 (0.85–1.08)	0.493	0.97 (0.85–1.11)	0.704
Q3 (17.5–20)	450/30 755	473 311	95	0.83 (0.73–0.94)	0.003	0.86 (0.75–0.99)	0.046
Q4 (20.5–30)	326/26 459	410 002	80	0.70 (0.61–0.80)	<0.001	0.83 (0.71–0.99)	0.034
*P* trend							0.010
Atrial fibrillation							
Q1 (6–14)	266/32 403	482 208	55	1.00 [reference]		1.00 [reference]	
Q2 (14.5–17)	257/33 713	517 112	50	1.04 (0.88–1.24)	0.635	1.06 (0.87–1.28)	0.547
Q3 (17.5–20)	212/30 755	475 797	45	0.95 (0.79–1.14)	0.564	0.94 (0.76–1.16)	0.553
Q4 (20.5–30)	194/26 459	411 730	47	1.05 (0.87–1.27)	0.573	1.10 (0.87–1.38)	0.418
*P* trend							0.725

Quartile 1 represents the least adherent to the Portfolio Diet, whereas quartile 4 represents the most adherence to the Portfolio Diet. Associations between Portfolio Diet and outcomes were determined by Cox proportional hazard models. Under/over energy reporters and those with baseline CVD were excluded from the analysis. Total CVD is a composite of incidence and death of CHD, stroke, heart failure, and coronary revascularization (coronary artery bypass grafting or percutaneous transluminal coronary angioplasty). CHD indicates coronary heart disease; CT, clinical trial; CVD, cardiovascular disease; HR, hazard ratio; OS, observational study; and Q, quartile.

* Model 1 adjusted for age (continuous), region (Northeast, South, Midwest, West), smoking (never, past, current), and study arm (hormone replacement therapy arm, dietary modification arm, calcium and vitamin D arm).

^†^ Model 2 adjusted for model 1+race/ethnicity (White, Black, Hispanic, Asian/Pacific Islander, Other [American Indian, Alaskan Native, other]), education (college or above, below college), marital status (presently married/other), hysterectomy history (yes/no), body mass index (continuous), physical activity (continuous), alcohol intake (>7 drinks/week, <7 drinks/week), energy intake (continuous), cancer status (yes/no), hypertension status (yes/no), diabetes mellitus status (yes/no), sodium intake (continuous), family history of CVD (yes/no), family history of diabetes mellitus (yes/no), hormone therapy use (never, past, current), cholesterol‐lowering medication use (yes/no).

**Table 4:** Prospective Association of the Portfolio Diet Score With Risk of Cardiovascular Disease Outcomes Among 70 506 Participants in the Observational Study of the Women's Health Initiative (1993–2017)

	Cases/Total	Person‐Years	Incidence Rate (Per 100 000 Person‐Years)	Model 1* (n=69 196)		Model 2^†^ (n=60 923)	
				HR (95% CI)	*P* Value	HR (95% CI)	*P* Value
Total CVD							
Q1 (6–14)	1721/16 472	222 515	773	1.00 [reference]		1.00 [reference]	
Q2 (14.5–17)	1910/19 350	274 964	694	0.89 (0.83–0.95)	<0.001	0.94 (0.88–1.01)	0.091
Q3 (17.5–20)	1654/18 297	264 916	624	0.79 (0.74–0.84)	<0.001	0.86 (0.78–0.93)	<0.001
Q4 (20.5–30)	1345/16 387	241 091	558	0.71 (0.66–0.76)	<0.001	0.85 (0.78–0.93)	<0.001
*P* trend							<0.001
CHD							
Q1 (6–14)	745/16 472	229 456	325	1.00 [reference]		1.00 [reference]	
Q2 (14.5–17)	752/19 350	283 605	265	0.79 (0.72–0.88)	<0.001	0.87 (0.78–0.97)	0.012
Q3 (17.5–20)	681/18 297	271 936	250	0.74 (0.66–0.82)	<0.001	0.81 (0.72–0.91)	<0.001
Q4 (20.5–30)	577/16 387	246 743	234	0.69 (0.61–0.77)	<0.001	0.82 (0.71–0.93)	0.003
*P* trend							0.002
Stroke							
Q1 (6–14)	496/16 472	230 524	215	1.00 [reference]		1.00 [reference]	
Q2 (14.5–17)	631/19 350	283 869	222	0.99 (0.88–1.20)	0.922	1.05 (0.92–1.19)	0.448
Q3 (17.5–20)	533/18 297	272 608	196	0.86 (0.76–0.97)	0.015	0.93 (0.81–1.07)	0.310
Q4 (20.5–30)	446/16 387	247 169	180	0.78 (0.69–0.89)	<0.001	0.92 (0.78–1.08)	0.320
*P* trend							0.224
Heart failure							
Q1 (6–14)	292/16 472	231 297	126	1.00 [reference]		1.00 [reference]	
Q2 (14.5–17)	300/19 350	285 357	105	0.88 (0.74–1.03)	0.114	0.88 (0.73–1.05)	0.156
Q3 (17.5–20)	233/18 297	273 961	85	0.71 (0.59–0.85)	<0.001	0.75 (0.62–0.92)	0.005
Q4 (20.5–30)	193/16 387	248 275	78	0.67 (0.56–0.81)	<0.001	0.80 (0.64–1.00)	0.053
*P* trend							0.009
Atrial fibrillation							
Q1 (6–14)	20/16 472	232 957	9	1.00 [reference]		1.00 [reference]	
Q2 (14.5–17)	24/19 350	287 170	8	0.89 (0.49–1.62)	0.704	1.33 (0.67–2.56)	0.401
Q3 (17.5–20)	14/18 297	275 243	5	0.57 (0.29–1.13)	0.108	0.87 (0.40–1.94)	0.764
Q4 (20.5–30)	18/16 387	249 464	7	0.80 (0.41–1.54)	0.510	1.33 (0.60–2.94)	0.484
*P* trend							0.749

Quartile 1 represents the least adherent to the Portfolio Diet, whereas quartile 4 represents the most adherence to the Portfolio Diet. Associations between Portfolio Diet and outcomes were determined by Cox proportional hazard models. Under/over energy reporters and those with baseline CVD were excluded from the analysis. Total CVD is a composite of incidence and death of CHD, stroke, heart failure, and coronary revascularization (coronary artery bypass grafting or percutaneous transluminal coronary angioplasty). CHD indicates coronary heart disease; CVD, cardiovascular disease; HR, hazard ratio; and Q, quartile.

* Model 1 adjusted for age (continuous), region (Northeast, South, Midwest, West), and smoking (never, past, current).

^†^ Model 2 adjusted for model 1+ethnicity (White, Black, Hispanic, Asian/Pacific Islander, Other [American Indian, Alaskan Native, other]), education (college or above, below college), marital status (presently married/other), hysterectomy history (yes/no), boyd mass index (continuous), physical activity (continuous), alcohol intake (>7 drinks/week, <7 drinks/week), energy intake (continuous), cancer status (yes/no), hypertension status (yes/no), diabetes mellitus status (yes/no), sodium intake (continuous), family history of CVD (yes/no), family history of diabetes mellitus (yes/no), hormone therapy use (never, past, current), cholesterol‐lowering medication use (yes/no).

### Sensitivity Analyses

The associations between the Portfolio Diet score and CVD outcomes remained similar in all sensitivity analyses (in the OS participants only [Table 4], and baseline diet only, excluding participants from the dietary modification trial, excluding CVD events within the first 3 years of follow‐up, excluding those with diabetes mellitus, and completing multiple imputation for missing covariate data [Table S4]). For HF, however, after excluding events diagnosed in the first 3 years, the association was slightly attenuated and no longer significant (Table S4). The association between the Portfolio Diet score based on the randomized clinical trials recommendations and CVD outcomes were attenuated and no longer significant for some outcomes; however, patterns were similar to our original a priori analysis (Table S3).

### Subgroup Analyses

The results remained largely consistent in each of the subgroup analyses, apart from effect modification by smoking status and CHD (Figures S1 through S5).

### Individual Component Analyses

When we individually assessed the 6 components of the Portfolio Diet score with the CVD outcomes, higher intakes of nuts, phytosterols, and MUFAs and lower intake of saturated fat sources had inverse associations with total CVD. Phytosterols and low saturated fat sources had inverse associations with CHD and phytosterols had inverse associations with stroke. Nuts also had an inverse association with HF (Table S5).

## Discussion

In this large prospective cohort study of US postmenopausal women, a higher Portfolio Diet score was associated with a 11% and 14% lower risk of total CVD and CHD, respectively, but no association was seen with stroke. These findings remained consistent across all sensitivity analyses, including when we excluded the WHI clinical trial participants, highlighting the robustness of our results. There was also a strong linear trend for greater adherence to the Portfolio Diet with total CVD and CHD. For our secondary analysis, there was an association of a 17% lower risk of HF with a higher Portfolio Diet score, but no association was seen with AF. The true benefits of the Portfolio Diet on CVD risk reduction, however, are likely underestimated in the current study.

### Interpretation of Results and Implications

These findings are consistent with the Portfolio Diet trial evidence assessing effects on intermediate risk factors for CVD. The Portfolio Diet has been shown to result in clinically meaningful reductions in the lipid targets for CVD prevention (LDL‐C, non‐high‐density lipoprotein cholesterol, ApoB), as well as CRP, with smaller reductions in blood pressure.^8^ In particular, LDL‐C, the primary risk factor that the Portfolio Diet was designed to reduce, is considered causal in the pathogenesis of atherosclerotic CVD based on evidence from cardiovascular outcomes trials involving 3 different classes of drugs (statins, ezetimibe, and PCSK9 inhibitors), Mendelian randomization studies and prospective cohorts.^27^ Our strongest finding of a 14% inverse association with CHD is consistent with these lines of evidence and closely reflects the predicted 10‐year CHD risk reduction of 13% estimated in our systematic review and meta‐analysis of the Portfolio Diet trials.^8^ The 0.73 mmol/L reduction in LDL‐C that corresponds to this 13% reduction in the Portfolio Diet trials is predicted by the regression line for the observed risk reduction per mmol/L of LDL‐C seen within the updated analyses of the CTT (Cholesterol Treatment Trialists) collaboration.^28^

We are unaware of other studies examining the association of a Portfolio Diet with CVD events. The individual components of the Portfolio Diet, however, have been associated with lower rates of CVD events in prospective cohorts. Systematic reviews and meta‐analyses have shown that consumption of legumes,^13^ dietary fiber including viscous fiber sources,^14^ nuts,^15^ and MUFAs^16^ are associated with reductions in CVD events, and consumption of foods high in saturated fat (such as red and processed meats) are associated with an increased risk of CVD.^17^ The inverse association of increasing phytosterol intake from natural sources with CVD risk in our study, however, was not shown in an earlier study.^29^

The Portfolio Diet also shows similar results to other recognized dietary patterns for CVD prevention, such as the Dietary Approaches to Stop Hypertension, vegetarian, Nordic, and Mediterranean diets, which share important overlap in core foods (nuts, legumes, whole grains, fruit/vegetable sources, and/or monounsaturated fat).^18^, ^30^, ^31^, ^32^, ^33^ Systematic reviews and meta‐analyses of prospective cohort studies and large individual cohort studies have shown the Dietary Approaches to Stop Hypertension diet is associated with a 20% (95% CI, 0.76–0.85 HRs) reduction in CVD and a 21% reduction (0.71–0.88) in CHD incidence,^30^ whereas Nordic and vegetarian diets are associated with 29% (0.65–0.78) and 22% (0.69–0.88) reductions in CVD and CHD mortality, respectively.^31^, ^33^ Similarly, the PREDIMED trial, a large randomized cardiovascular outcomes trial of the effect of a Mediterranean diet supplemented with either extra virgin olive oil or nuts compared with a low‐fat diet, found reductions in major vascular events of 31% (0.53–0.91) and 28% (0.54–0.95), respectively.^18^

Dietary patterns have also shown similar results specifically within the WHI. Higher adherence to the Healthy Eating Index 2010, Alternative Healthy Eating Index 2010, Alternate Mediterranean and Dietary Approaches to Stop Hypertension diets have been associated with 18% to 26% lower CVD mortality risk in the OS participants,^34^ which falls within the 95% CIs (HR, 0.85; 95% CI, 0.78–0.93) of our findings for total CVD comparing lowest to highest adherence of the Portfolio Diet score in these participants. The 30% reduction in HF associated with higher adherence to the Alternative Healthy Eating Index^24^ also falls within the 95% CIs (HR, 0.80; 95% CI, 0.64–0.99) of our findings for the Portfolio Diet in the OS participants.

Unlike some other dietary patterns, adherence to the Portfolio Diet was not associated with a reduction in stroke in our study. Both the Mediterranean and Dietary Approaches to Stop Hypertension diets have shown inverse associations with stroke.^30^, ^32^ Although the Portfolio Diet resulted in a reduction in blood pressure in the randomized trials,^8^ this effect may not be strong enough to translate into an association with lower stroke risk, given that the reductions were small and hypertension is the most important risk factor for stroke.^35^ The larger reductions in LDL‐C and other lipid targets, as well as CRP, may be more relevant for the inverse associations seen with CHD and total CVD than with stroke. AF is also a major risk factor for stroke,^36^ and we did not observe a significant association with lower AF risk in our study.

These findings highlight the plant‐based Portfolio Diet as another dietary therapeutic approach for CVD prevention, alongside other dietary patterns recommended for CVD prevention. As adherence is one of the most critical determinants for attaining the benefits of any diet, as recognized by cardiovascular clinical practice guidelines,^9^ the Portfolio Diet may best fit with the values and preferences of some patients and allow them to achieve the greatest adherence long term. The Portfolio Diet also has a small ecological footprint, emphasizing plant‐based components with low environmental impact (eg, legumes, oats, barley, temperate fruit, etc).^37^, ^38^ Given increasing public concerns regarding ethical and environmental impact of food,^39^, ^40^ healthcare professionals will likely have more patients interested in this dietary pattern.

### Strengths and Limitations

Strengths of our study include the prospective cohort design, large sample size, and long follow‐up for incident CVD events. Nevertheless, this study does have limitations. First, our study included only 1 or 2 assessments of diet, and diet was self‐reported. Second, the population included health‐conscious postmenopausal women and therefore the results may not be generalizable to men or other populations; however, the Portfolio Diet trials were conducted in both men and postmenopausal women and benefits were seen in both sexes. Third, causation cannot be established because of the observational design, and residual confounding also cannot be ruled out. Lastly, consumption of many of the Portfolio Diet components remained low, particularly plant protein and MUFAs, even in the top quintiles. A few of the Portfolio Diet foods, such as some viscous fiber sources (eg, barley), were also not included on the FFQ. This finding was further highlighted in our post hoc sensitivity analysis where we created a Portfolio Diet score based on the recommendations of the Portfolio Diet trials. No participants in the WHI received the maximum amount of points possible, and maximum points suggested ≈50% adherence to the trial recommendations, with an average estimated adherence of ≈22%. These adherence estimations are, however, likely underestimated given the nature of FFQs and their inability to determine absolute intake of diets. Taken together, we expect that the associations are likely underestimated, and a stronger association with CVD events may be seen with greater consumption of the Portfolio Diet components. This low adherence reflects an important opportunity for individuals to achieve cardiovascular benefits of the Portfolio Diet. Typical dietary patterns in North America and Europe do not meet the targets for plant protein, viscous fiber, nuts, phytosterols, and MUFAs of the Portfolio Diet,^41^, ^42^, ^43^, ^44^, ^45^, ^46^ and therefore public health initiatives that focus on the components of the diet may improve cardiovascular outcomes globally. It will be of great interest to apply this Portfolio Diet score to other populations, particularly in those that consume greater amounts of the diet components, to assess if similar or stronger associations with incident CVD events are found.

## Conclusions

Greater adherence to the plant‐based Portfolio Diet score was significantly associated with lower risk of total CVD, CHD, and HF in postmenopausal women. These findings provide the strongest evidence to date on the long‐term benefits of a Portfolio Diet in the primary prevention of CVD, although our Portfolio Diet score needs to be assessed in other cohorts/populations to confirm these findings. Evidence from randomized trials with clinical CVD events is also needed. In this regard, we await the results of the PortfolioEX trial (ClinicalTrials.gov Identifier: NCT02481466) of the effect of the Portfolio Diet plus exercise on a surrogate marker of atherosclerotic CVD risk (magnetic resonance imaging of atherosclerosis [plaque volume]). In the meantime, our results support the Portfolio Diet as another therapeutic dietary approach for managing CVD risk that fits with current guidelines emphasizing plant‐based diets.

## Sources of Funding

The Women's Health Initiative (WHI) was funded by the National Heart, Lung, and Blood Institute, National Institutes of Health, and U.S. Department of Health and Human Services through contracts HHSN268201600018C, HHSN268201600002C, HHSN268201600003C, HHSN268201600004C, and R01DK125403 (SL). Glenn was supported by the Nora Martin Fellowship in Nutritional Sciences, the Banting & Best Diabetes Centre Tamarack Graduate Award in Diabetes Research, the Peterborough K.M. Hunter Charitable Foundation Graduate Award and an Ontario Graduate Scholarship. Sievenpiper was funded by a Diabetes Canada Clinician Scientist Award. Lo was supported by Start‐up Fund for RAPs under the Strategic Hiring Scheme (Grant number: BD8H). Funders had no role in the study design, the collection, analysis and interpretation of data, the writing of the report, and the decision to submit the article for publication.

## Disclosures

A.J.G. received funding from the Nora Martin Fellowship in Nutritional Sciences, Banting & Best Diabetes Centre Tamarack Graduate Award in Diabetes Research, the Peterborough K.M. Hunter Charitable Foundation Graduate Award, and the Ontario Graduate Scholarship. She has received consulting fees from Solo GI Nutrition and has received an honorarium from the Soy Nutrition Institute. D.J.A.J. has received research grants from Saskatchewan & Alberta Pulse Growers Associations, the Agricultural Bioproducts Innovation Program through the Pulse Research Network, the Advanced Foods and Material Network, Loblaw Companies Ltd., Unilever Canada and Netherlands, Barilla, the Almond Board of California, Agriculture and Agri‐food Canada, Pulse Canada, Kellogg's Company, Canada, Quaker Oats, Canada, Procter & Gamble Technical Centre Ltd., Bayer Consumer Care, Springfield, NJ, Pepsi/Quaker, International Nut & Dried Fruit Council (INC), Soy Foods Association of North America, the Coca‐Cola Company (investigator initiated, unrestricted grant), Solae, Haine Celestial, the Sanitarium Company, Orafti, the International Tree Nut Council Nutrition Research and Education Foundation, the Peanut Institute, Soy Nutrition Institute (SNI), the Canola and Flax Councils of Canada, the Calorie Control Council, the Canadian Institutes of Health Research (CIHR), the Canada Foundation for Innovation (CFI)and the Ontario Research Fund (ORF). He has received in‐kind supplies for trials as a research support from the Almond board of California, Walnut Council of California, the Peanut Institute, Barilla, Unilever, Unico, Primo, Loblaw Companies, Quaker (Pepsico), Pristine Gourmet, Bunge Limited, Kellogg Canada, WhiteWave Foods. He has been on the speaker's panel, served on the scientific advisory board and/or received travel support and/or honoraria from 2020 China Glycemic Index (GI) International Conference, Atlantic Pain Conference, Academy of Life Long Learning, the Almond Board of California, Canadian Agriculture Policy Institute, Loblaw Companies Ltd, the Griffin Hospital (for the development of the NuVal scoring system), the Coca‐Cola Company, Epicure, Danone, Diet Quality Photo Navigation (DQPN), Better Therapeutics (FareWell), Verywell, True Health Initiative (THI), Heali AI Corp, Institute of Food Technologists (IFT), Soy Nutrition Institute (SNI), Herbalife Nutrition Institute (HNI), Saskatchewan & Alberta Pulse Growers Associations, Sanitarium Company, Orafti, the International Tree Nut Council Nutrition Research and Education Foundation, the Peanut Institute, Herbalife International, Pacific Health Laboratories, Nutritional Fundamentals for Health (NFH), Barilla, Metagenics, Bayer Consumer Care, Unilever Canada and Netherlands, Solae, Kellogg, Quaker Oats, Procter & Gamble, Abbott Laboratories, Dean Foods, the California Strawberry Commission, Haine Celestial, PepsiCo, the Alpro Foundation, Pioneer Hi‐Bred International, DuPont Nutrition and Health, Spherix Consulting and WhiteWave Foods, the Advanced Foods and Material Network, the Canola and Flax Councils of Canada, Agri‐Culture and Agri‐Food Canada, the Canadian Agri‐Food Policy Institute, Pulse Canada, the Soy Foods Association of North America, the Nutrition Foundation of Italy (NFI), Nutra‐Source Diagnostics, the McDougall Program, the Toronto Knowledge Translation Group (St. Michael's Hospital), the Canadian College of Naturopathic Medicine, The Hospital for Sick Children, the Canadian Nutrition Society (CNS), the American Society of Nutrition (ASN), Arizona State University, Paolo Sorbini Foundation and the Institute of Nutrition, Metabolism and Diabetes. He received an honorarium from the United States Department of Agriculture to present the 2013 W.O. Atwater Memorial Lecture. He received the 2013 Award for Excellence in Research from the International Nut and Dried Fruit Council. He received funding and travel support from the Canadian Society of Endocrinology and Metabolism to produce mini cases for the Canadian Diabetes Association (CDA). He is a member of the International Carbohydrate Quality Consortium (ICQC). His wife, Alexandra L Jenkins, is a director and partner of INQUIS Clinical Research for the Food Industry, his 2 daughters, Wendy Jenkins and Amy Jenkins, have published a vegetarian book that promotes the use of the foods described here, The Portfolio Diet for Cardiovascular Risk Reduction (Academic Press/Elsevier 2020 ISBN:978‐0‐12‐810510‐8)and his sister, Caroline Brydson, received funding through a grant from the St. Michael's Hospital Foundation to develop a cookbook for one of his studies. A.J.H. received independent investigator‐initiated research funding from Dairy Farmers of Canada. C.W.C.K. has received research support from the Advanced Foods and Materials Network, Agricultural Bioproducts Innovation Program through the Pulse Research Network, Agriculture and Agri‐Food Canada, Almond Board of California, Barilla, Calorie Control Council, Canadian Institutes of Health Research, Canola Council of Canada, The International Tree Nut Council Nutrition Research & Education Foundation, Kellogg, Loblaw Companies Ltd., Pulse Canada, Saskatchewan Pulse Growers, and Unilever. He has received consultant fees from American Pistachio Growers; speaker fees from Tate & Lyle and The WhiteWave Foods Company; and travel funding from Sabra Dipping Company, Tate & Lyle, International Tree Nut Council Research & Education Foundation, California Walnut Commission, Sun‐Maid, The Peanut Institute, General Mills, Oldways Foundation and International Nut and Dried Fruit Council Foundation. He is on the Clinical Practice Guidelines Expert Committee for Nutrition Therapy of the European Association for the Study of Diabetes. He is a member of the International Carbohydrate Quality Consortium, Secretary of the Diabetes and Nutrition Study Group of the European Association for the Study of Diabetes, and a director of the Toronto 3D Knowledge Synthesis and Clinical Trials foundation. J.L.S. has received research support from the Canadian Foundation for Innovation, Ontario Research Fund, Province of Ontario Ministry of Research and Innovation and Science, Canadian Institutes of health Research (CIHR), Diabetes Canada, PSI Foundation, Banting and Best Diabetes Centre (BBDC), American Society for Nutrition (ASN), INC International Nut and Dried Fruit Council Foundation, National Dried Fruit Trade Association, National Honey Board (the U.S. Department of Agriculture [USDA] honey “Checkoff” program), International Life Sciences Institute (ILSI), Pulse Canada, Quaker, The United Soybean Board (the USDA soy “Checkoff” program), The Tate and Lyle Nutritional Research Fund at the University of Toronto, The Glycemic Control and Cardiovascular Disease in Type 2 Diabetes Fund at the University of Toronto (a fund established by the Alberta Pulse Growers), and The Nutrition Trialists Fund at the University of Toronto (a fund established by an inaugural donation from the Calorie Control Council). He has received in‐kind food donations to support a randomized controlled trial from the Almond Board of California, California Walnut Commission, Peanut Institute, Barilla, Unilever/Upfield, Unico/Primo, Loblaw Companies, Quaker, Kellogg Canada, WhiteWave Foods/Danone, and Nutrartis. He has received travel support, speaker fees and/or honoraria from Diabetes Canada, Dairy Farmers of Canada, FoodMinds LLC, International Sweeteners Association, Nestlé, Pulse Canada, Canadian Society for Endocrinology and Metabolism (CSEM), GI Foundation, Abbott, General Mills, Biofortis, ASN, Northern Ontario School of Medicine, INC Nutrition Research & Education Foundation, European Food Safety Authority (EFSA), Comité Européen des Fabricants de Sucre (CEFS), Nutrition Communications, International Food Information Council (IFIC), Calorie Control Council, and Physicians Committee for Responsible Medicine. He has or has had ad hoc consulting arrangements with Perkins Coie LLP, Tate & Lyle, Wirtschaftliche Vereinigung Zucker e.V., Danone, and Inquis Clinical Research. He is a member of the European Fruit Juice Association Scientific Expert Panel and Soy Nutrition Institute (SNI) Scientific Advisory Committee. He is on the Clinical Practice Guidelines Expert Committees of Diabetes Canada, European Association for the study of Diabetes (EASD), Canadian Cardiovascular Society (CCS), and Obesity Canada/Canadian Association of Bariatric Physicians and Surgeons. He serves or has served as an unpaid scientific advisor for the Food, Nutrition, and Safety Program (FNSP) and the Technical Committee on Carbohydrates of ILSI North America. He is a member of the International Carbohydrate Quality Consortium (ICQC), Executive Board Member of the Diabetes and Nutrition Study Group (DNSG) of the EASD, and Director of the Toronto 3D Knowledge Synthesis and Clinical Trials foundation. His wife is an employee of AB InBev. There are no other relationships or activities that could appear to have influenced the submitted work.

## Supporting information

Data S1
Appendix S1
Tables S1–S5
Figures S1–S5
References 47, 48, 49, 50, 51, 52, 53, 54, 55, 56, 57
