# Supplementary material for: Relationship Between a Plant‐Based Dietary Portfolio and Risk of Cardiovascular Disease: Findings From the Women's Health Initiative Prospective Cohort Study
Source: J Am Heart Assoc. 2021 Aug 4;10(16):e021515. doi: 10.1161/JAHA.121.021515 (PMC8475059; doi:10.1161/JAHA.121.021515)
Supplement: Supplementary file 1 — Data S1 Appendix S1 Tables S1–S5 Figures S1–S5 References 47, 48, 49, 50, 51, 52, 53, 54, 55, 56, 57 [file JAH3-10-e021515-s001.pdf]

SUPPLEMENTAL MATERIAL

Data S1.

Supplemental Methods

Study design:
At baseline, participants reported information on demographic factors, health behaviors, and medical histories using self-administered questionnaires. At the baseline clinic visit, trained staff measured weight and height.

Dietary Assessment:
Diet was assessed by a 122-item modified Block food frequency questionnaire (FFQ) developed and validated for the WHI population[22]. The FFQ was administered at baseline for all participants, and again at year 3 for observational study participants. The FFQ asked questions about frequency of food consumption over the previous 3 months, with 19 adjustment items primarily related to fat intake, and 4 summary questions[22]. The frequency questions included predefined responses that ranged from "never or less than once per month" to "2 or more times per day" for foods and "6 or more times per day" for drinks. Small, medium or large portion sizes were assessed as compared to specified medium size portions. Pictures were provided to help with portion size estimation. The nutrient database for the WHI FFQ uses the Nutrition Data Systems for Research (NDS-R, version 2006), University of Minnesota Nutrition Coordinating Center, Minneapolis, MN) food and nutrient database[47].

Portfolio Diet Score development:
We previously developed a diet score for assessing adherence to the Portfolio Diet. Food items recommended in the Portfolio Diet were extracted from the WHI FFQ and categorized into the 6 components of the Portfolio Diet (refer to Tables 2 and S1). For most components, servings/day were summed over all consumed food items in each component for every participant. More points were given to participants with higher intakes of foods recommended in the Portfolio Diet, whereas less points were given to participants with higher intakes of foods not recommended in the Portfolio Diet. These points were given for each of the 6 dietary components by splitting the components into quintiles: those in the highest quintile of foods recommended (such as nuts) received 5 points and those in the lowest quintile received 1 point. Reverse scoring was done for those with foods not recommended (such as foods high in saturated fat), as those with the highest intake (quintile 5) received 1 point, and those with the lowest intake (quintile 1) received 5 points. The total points were then added for each participant, resulting in a score range between 6 and 30, with higher scores indicating higher adherence to the Portfolio diet. Food items from the FFQ and included in our Portfolio Diet score are in Table S1.

Plant sterols was the only score component based on mg/day. All other components are food-based in servings/day. The WHI FFQ compositional database did not have plant sterols available as a nutrient variable for their FFQ. Therefore, we developed a plant sterol database (mg/d) based on literature values for ~350 foods and created recipes to match the 122 food items in the WHI FFQ. A number of data sources were used, including European databases: the Finnish Food Composition Database[48], and the database used in the European Prospective Cohort into Cancer (EPIC) cohort[29]; the United States Department of Agriculture (USDA)[49] and other literature[43,50-53]. We created recipes and determined plant sterol values for foods in the WHI FFQ that did not have a plant sterol values available in the literature, using the ESHA Research Food Processor SQL: Nutrition Analysis and Fitness Program (Copyright 2012, ESHA Research).

Additionally, our approach for determining the Portfolio Diet score was based on several factors. First, we chose population-based intake cut-offs rather than the absolute amounts of the Portfolio Diet components from the clinical trials, as we would not expect a substantial portion of the population to follow the Portfolio Diet, particularly because the FFQ data in WHI were collected before the Portfolio Diet trials were conducted and published. FFQs are also not designed to measure absolute intake, and are better suited for ranking individuals, therefore a population-based cut-off is more appropriate. In addition, we primarily chose a food-based approach rather than a nutrient-based approach as this reflects how the Portfolio Diet is implemented in clinical practice, food-based recommendations may be easier to interpret, and they are more suitable and transferable to multiple FFQs. We did not weight the six components differently as although evidence has shown that a low saturated fat intake may lower LDL-C more than the other components, weighting the components differently did not change the results of preliminary work we had done when developing the Portfolio Diet score. We therefore believe our current method

assesses greater adherence to the Portfolio Diet, while still allowing for adequate variation in the scores to examine associations with disease outcomes in prospective cohorts.

Outcomes:
Heart failure outcome included in the study is the original WHI outcome, referred to as congestive heart failure (CHF). Subtypes based on reduced/preserved ejection fraction rate were not recorded, and therefore not assessed in this analysis. Atrial fibrillation outcomes were collected in WHI extension 2 and beyond and is only adjudicated for the Medical Record Cohort.

Covariates:
Additional information on baseline measurements are included as follows:

Physical activity: Women reported the frequency, duration, and intensity of recreational physical activity, including walking, mild, moderate, and strenuous activity[21] using a validated physical activity questionnaire. From these data, metabolic equivalents of physical activities in metabolic equivalents-hours/wk (kcal/wk per kg) were computed[54].

Medical history: In the medical history questionnaire, women were asked (yes/no), "Has a doctor told you that you have, or have you had high cholesterol requiring pills?" for baseline lipid-lowering medication use. Baseline hypertensive status was self-selected as "never hypertensive," "untreated hypertensive," or "treated hypertensive." Subjects were also asked, "Has a doctor ever told you that you had heart problems, problems with your blood circulation, or blood clots?" Previous validation studies have found self-report of CVD at baseline in the WHI to be reliable[55,56]. For baseline diabetes status, participants were asked if a physician had ever told them they had "sugar diabetes or high blood sugar" when they were not pregnant, and about treatment with insulin or oral diabetes medications. Diabetes was defined as a confirmatory answer to the above question or reported use of medication to treat diabetes. A validation study of the accuracy of self-reported diabetes was found to be reliable[57].

Body weight: Trained and certified WHI clinical staff measured height and weight using standardized procedures. Weight was measured using a calibrated balance-beam scale and height using a fixed stadiometer. From these measurements, body mass index (BMI) was calculated as weight in kilograms divided by height in meters squared[19].

Statistical Analyses:
Time to event: The time to event was measured as the number of days since enrollment to the first occurrence of a cardiovascular event. Otherwise, participants were censored at the time of a woman's last documented follow-up contact, whether due to loss of follow-up, non-cardiovascular death or end of study.

Cumulative average: For participants who completed two food frequency questionnaires (FFQs, at baseline and year 3 in the observational study (OS)) the cumulative average of the Portfolio Diet score was related to CVD outcomes. Therefore, if a participant in the OS study had a CVD event before the year 3 FFQ was completed, the baseline Portfolio Diet score was related to the CVD outcomes in our analyses. Otherwise, CVD outcomes were related to the average of the diet scores from both FFQs. The person correlation coefficient between the baseline and year 3 Portfolio Diet scores was 0.63 (<0.05).

Short list of WHI Investigators

Program Office: (National Heart, Lung, and Blood Institute, Bethesda, Maryland) Jacques Rossouw, Shari Ludlam, Joan McGowan, Leslie Ford, and Nancy Geller.

Clinical Coordinating Center: (Fred Hutchinson Cancer Research Center, Seattle, WA) Garnet Anderson, Ross Prentice, Andrea LaCroix, and Charles Kooperberg.

Investigators and Academic Centers:
(Brigham and Women's Hospital, Harvard Medical School, Boston, MA) JoAnn E. Manson; (MedStar Health Research Institute/Howard University, Washington, DC) Barbara V. Howard; (Stanford Prevention Research Center, Stanford, CA) Marcia L. Stefanick; (The Ohio State University, Columbus, OH) Rebecca Jackson; (University of Arizona, Tucson/Phoenix, AZ) Cynthia A.Thomson; (University at Buffalo, Buffalo, NY) Jean Wactawski-Wende; (University of Florida, Gainesville/Jacksonville, FL) Marian Limacher; (University of Iowa, Iowa City/Davenport, IA) Jennifer Robinson; (University of Pittsburgh, Pittsburgh, PA) Lewis Kuller; (Wake Forest University School of Medicine, Winston-Salem, NC) Sally Shumaker; (University of Nevada, Reno, NV) Robert Brunner; (University of Minnesota, Minneapolis, MN) Karen L. Margolis Women's Health Initiative Memory Study:(Wake Forest University School of Medicine, Winston-Salem, NC) Mark Espeland.

For a list of all the investigators who have contributed to WHI science, please visit: https://www.whi.org/researchers/Documents%20%20Write%20a%20Paper/WHI%20Investigator%20Long%20List.pdf

Table S1. Key Characteristics between included and excluded participants.

Mean (SD)/ No. (%)	Included	Excluded*
Number of participants	123,330	5,884
Time-to-event/censored in years	15.3 (5.67)	13.1 (5.97)
Age (years)	62.6 (7.14)	63.4 (7.47)
BMI (kg/m^2)	27.8 (5.83)	27.9 (6.02)
Recreational physical activity (MET-hour/week)	12.8 (13.9)	11.9 (14.7)
Dietary energy (kcal/day)	1642 (640.5)	1252 (1552)
Region in the U.S		
Northeast	28491 (23.1)	1030 (18.4)
South	31376 (25.4)	1912 (34.2)
Midwest	27106(22.0)	1007 (18.0)
West	36357 (29.5)	1650 (29.5)
Race/ethnicity		
White	103284 (84.0)	3649 (65.5)
African American	10118 (8.2)	1061 (19.0)
Hispanic	4875 (4.0)	567 (10.2)
Asian	3390 (1.1)	205 (1.6)
Alcoholic Drinks		
>7 drinks/week	3846 (11.8)	342 (6.5)
Sodium intake (mg/day)	2742 (1152)	2131 (2870)
Hormone therapy use		
Never	40050 (33.5)	2201 (40.0)
Past	26594 (22.2)	1283 (23.3)
Current	52953 (44.3)	2017 (36.4)
Hysterectomy ever	49172 (40.0)	2428 (43.4)
Treated high cholesterol	13466 (11.6)	765 (14.7)
History of hypertension	36629 (30.0)	1731 (32.3)
History of cancer	10512 (8.6)	506 (9.0)
Family history diabetes	38664 (31.5)	1802 (34.0)
Family history of CVD	80129 (65.0)	3256 (55.3)
Self-reported diabetes	5741 (4.7)	394 (7.0)
Smoking status		
Never	63331 (51.4)	2266 (57.3)
Past	51578 (41.8)	1323 (33.5)
Current	8421 (6.8)	365 (9.23)
Education: college or above	83887 (68.5)	3086 (55.9)
Marital status: present relationship	78403 (63.9)	2924 (52.8)
HRT arm		
Not randomized	101653 (82.4)	4528 (80.9)
E-alone	3987 (3.2)	215 (3.8)
E-alone control	4098 (3.3)	247 (4.4)
E+P intervention	6972 (5.7)	305 (5.5)
E+P control	6620 (5.4)	304 (5.3)
DM arm		
Not randomized	84243 (68.3)	4990 (89.1)
Intervention	15588 (12.6)	251 (4.5)
Control	23499 (19.1)	358 (6.7)
CaD arm		
Not randomized	93911 (75.2)	4822 (86.1)
Intervention	14774 (12.0)	406 (7.25)
Control	14645 (11.9)	371 (6.63)

BMI, body mass index; CaD, calcium and vitamin D; CVD, cardiovascular disease; DM, dietary modification; E-alone, estrogen-alone; E+P, estrogen plus progestin;
HRT, hormone replacement therapy; Kcal, kilocalories; MET, metabolic equivalents; Q, quartile**;** SD, standard deviation; U.S., United States.
*A combination of 3,540 participants who had implausible energy intake and 2,344 who had missing covariates for model 1 (only smoking data was missing in this case) and missing time to event data.

Table S2. Full line items from WHI FFQ used to calculate the Portfolio Diet score primarily based on servings/day.

**Component**	
Plant protein	(1) Soy milk as beverage; (2) green or English peas; (3) refried beans, (4) all other beans such as baked beans, lima beans, black-eyed peas and chili without meat; (5) tofu and textured vegetable products; (6) bean soups such as pea, lentil, black bean, potajes
Viscous fiber	(1) Oranges, grapefruit & tangerines; (2) apples & pears; (3) strawberries & kiwi*; (4) summer squash, zucchini, nopales & okra†; (5) cooked cereals & grits‡
Nuts	(1) Peanut butter, peanuts, other nuts and seeds
Plant sterols	Estimated from all foods on FFQ (mg/day)
MUFAs	(1) Olive or canola oil as spread or added to food; (2) avocado & guacamole, including added to mixed dishes
Saturated fat	(1) Whole milk as beverage; (2) ground meat including hamburgers, meatloaf & picadillo; (3) beef, pork and lamb as a main dish, such as steak, roast and ham; (4) beef, pork and lamb as a sandwich (steak sandwich, BBQ sandwich); (5) Liver, including chicken liver and other organs; (6) Gravies made with meat drippings; (7) Lunch meat such as ham, turkey and other special lean meats; (8) All other lunch meat such as bologna, salami, Spam®, potted and canned meat; (9) Hot dogs, chorizo, and other sausage such as bratwurst; (10) eggs; (11) bacon, breakfast sausage and scrapple; (12) all other cheeses, such as cheddar, Swiss, or cream cheese; (13) ice cream; (14) fried chicken; (15) chicken or turkey (with skin only); (16) butter as spread, or added to food or cereal

*Half of question amount assumed to be strawberries; †One quarter of question amount assumed to be okra; ‡Half of cooked cereal question amount assumed to be oats.

Table S3. Sensitivity analyses of the association of the Portfolio Diet score based on RCT recommendations and cardiovascular outcomes.

		**TOTAL CVD**		**CHD**		**STROKE**		**HEART FAILURE**		**ATRIAL FIBRILLATION**	
		HR (95% CI)	P value	HR (95% CI)	P value	HR (95% CI)	P value	HR (95% CI)	P value	HR (95% CI)	P value
Portfolio diet score (0-7 points)		0.96 (0.94-0.99)	<0.01	0.96 (0.93-1.00)	0.05	0.94 (0.90-0.99)	0.01	0.91 (0.86-0.97)	<0.01	1.02 (0.93-1.13)	0.61
Portfolio diet Score tertiles*	N										
Q1 (0-2 points) 0-14% adherent	60,097	1.00 [reference]		1.00 [reference]		1.00 [reference]		1.00 [reference]		1.00 [reference]	
Q2 (3 points) 22% adherent	61,094	0.94 (0.90-0.99)	0.01	0.95 (0.89-1.02)	0.17	0.91 (0.84-0.99)	0.02	0.90 (0.80-1.01)	0.07	0.99 (0.84-1.18)	0.98
Q3 (4-7 points) 29-50% adherent	4,198	0.90 (0.80-1.00)	0.05	0.87 (0.74-1.05)	0.18	0.94 (0.78-1.13)	0.52	0.79 (0.57-1.08)	0.14	1.13 (0.75-1.71)	0.56

CHD, coronary heart disease; CVD, cardiovascular disease; HR, hazard ratio.

*Tertiles used instead of quartiles as per previous analysis based on range of 0-7 points (original analysis was 6-30).

The hazard ratios (HRs) are for assessing the Portfolio Diet score as a continuous exposure (0-7 points) and for comparing participants in Q1 (low adherence) to Q3 (high adherence) to the Portfolio Diet components based on recommendations from the Portfolio Diet RCTs (50g plant protein, 45g nuts, 20g viscous fiber, 2g plant sterols, 45g MUFAs, <7% energy from saturated fat and <200mg cholesterol per day). The scoring system to assess adherence to the Portfolio Diet score was based on 0-14 points (2 points for meeting previously listed 7 targets, 1 point for meeting half, and 0 points for less than half). Maximum score obtained was 7 points, therefore, 50% maximum adherence to the Portfolio Diet recommendations from the RCTs, with an average 22% adherence. Adherence to plant protein, viscous fiber and MUFAs was based on estimations of grams/servings for most commonly consumed foods in this category (plant protein was lentils/beans & green peas, viscous fiber was apples & oranges, and MUFAs were avocados). The remaining components were based on grams/day from the entire day as estimated from the FFQ.

The Cox regression models were adjusted for age, ethnicity, education, marital status, hysterectomy history, BMI, physical activity, smoking, alcohol intake, region in the U.S, study arm, energy intake, cancer status, hypertension status, diabetes status, sodium intake, family history of CVD, family history of diabetes, hormone therapy use, cholesterol lowering medication use.
Under/over energy reporters and those with baseline CVD were excluded from the analysis.

Table S4. Sensitivity analyses of the association of the Portfolio Diet score and cardiovascular outcomes.

	**Baseline Portfolio Diet Score only**		**Without Dietary Modification Participants**		**Removing those diagnosed in first 3 years**		**Excluding those with diabetes diagnosis**		**Multiple imputation of missing covariates**	
	HR (95% CI)	P value	HR (95% CI)	P value	HR (95% CI)	P value	HR (95% CI)	P value	HR (95% CI)	P value
**TOTAL CVD**										
Cases/total n	13,365/ 104,894		8,990/ 73,442		11,240/ 103,098		11,962/ 100,149		13,558/ 125,389	
Portfolio Score by Quartiles										
Q1	1.00 [reference]		1.00 [reference]		1.00 [reference]		1.00 [reference]		1.00 [reference]	
Q2	0.98 (0.93-1.03)	0.38	0.95 (0.89-1.01)	0.10	0.98 (0.92-1.03)	0.38	0.97 (0.92-1.02)	0.23	0.95 (0.91-0.99)	0.025
Q3	0.94 (0.87-0.97)	0.035	0.86 (0.81-0.92)	<0.001	0.92 (0.87-0.98)	0.006	0.91 (0.86-0.96)	0.001	0.89 (0.85-0.94)	<0.001
Q4	0.89 (0.84-0.95)	0.001	0.86 (0.80-0.93)	<0.001	0.88 (0.82-0.94)	<0.001	0.88 (0.82-0.94)	<0.001	0.86 (0.81-0.91)	<0.001
P trend		<0.001		<0.001		<0.001		<0.001		<0.001
**CHD**										
Cases/total n	5,640/ 104,894		3,810/ 72,442		4,924/ 104,302		5,006/ 100,152		5,739/ 125,389	
Portfolio Score by Quartiles										
Q1 (6-14)	1.00 [reference]		1.00 [reference]		1.00 [reference]		1.00 [reference]		1.00 [reference]	
Q2 (14.5-17)	0.96 (0.89-1.04)	0.29	0.91 (0.82-0.99)	0.04	0.92 (0.84-1.00)	0.05	0.90 (0.83-0.98)	0.01	0.89 (0.82-0.95)	0.001
Q3 (17.5-20)	0.89 (0.92-0.97)	<0.009	0.82 (0.74-0.91)	<0.001	0.85 (0.78-0.93)	<0.001	0.84 (0.77-0.92)	<0.001	0.84 (0.78-0.91)	<0.001
Q4 (20.5-30)	0.91 (0.83-0.99)	0.048	0.85 (0.76-0.95)	0.005	0.86 (0.78-0.96)	0.005	0.85 (0.77-0.94)	0.002	0.83 (0.76-0.91)	<0.002
P trend		<0.001		0.001		0.001		0.001		<0.001
**STROKE**										
Cases/total n	4,440/ 104,894		2,960/ 73,441		3,807/ 104,390		4,039/ 100,151		4,451/ 125,892	
Portfolio Score by Quartiles										
Q1 (6-14)	1.00 [reference]		1.00 [reference]		1.00 [reference]		1.00 [reference]		1.00 [reference]	
Q2 (14.5-17)	1.05 (0.96-1.15)	0.29	0.99 (0.89-1.10)	0.83	1.03 (0.93-1.14)	0.56	1.06 (0.96-1.16)		1.02 (0.95-1.11)	0.55

Q3 (17.5-20)	0.99 (0.90-1.09)	0.88	0.88 (0.78-0.99)	0.04	0.99 (0.89-1.10)	0.81	0.98 (0.89-1.09)		0.95 (0.88-1.03)	0.23
Q4 (20.5-30)	0.97 (0.87-1.08)	0.54	0.90 (0.79-1.03)	0.11	0.97 (0.86-1.09)	0.64	0.99 (0.88-1.10)		0.94 (0.85-1.04)	0.22
P trend		0.50		0.06		0.55		0.63		0.13
**HEART FAILURE**										
Cases/total n	1,907/ 104,894		1,303/ 73,440		1,467/ 104,519		1,557/ 100,150		1,946/ 125,389	
Portfolio Score by Quartiles										
Q1 (6-14)	1.00 [reference]		1.00 [reference]		1.00 [reference]		1.00 [reference]		1.00 [reference]	
Q2 (14.5-17)	0.94 (0.82-1.07)	0.34	0.94 (0.80-1.10)	0.42	0.96 (0.83-1.12)	0.64	0.92 (0.80-1.07)	0.29	0.98 (0.87-1.11)	0.79
Q3 (17.5-20)	0.92 (0.80-1.06)	0.26	0.79 (0.66-0.94)	0.008	0.86 (0.73-1.02)	0.09	0.86 (0.73-1.01)	0.07	0.88 (0.77-1.01)	0.06
Q4 (20.5-30)	0.79 (0.66-0.92)	0.005	0.81 (0.66-0.99)	0.035	0.85 (0.70-1.03)	0.10	0.86 (0.72-1.04)	0.11	0.79 (0.68-0.92)	0.002
P trend		0.010		0.005		0.05		0.05		0.001
**ATRIAL FIBRILLATION**[*]										
Cases/total n	929/ 104,894		634/ 73,442		0/ 104,894		874/ 100,152		935/ 125,389	
Portfolio Score by Quartiles										
Q1 (6-14)	1.00 [reference]		1.00 [reference]		NA	NA	1.00 [reference]		1.00 [reference]	
Q2 (14.5-17)	1.06 (0.87-1.27)	0.61	1.16 (0.92-1.47)	0.20	NA	NA	1.05 (0.86-1.29)	0.61	1.04 (0.87-1.23)	0.69
Q3 (17.5-20)	0.95 (0.77-1.17)	0.62	0.97 (0.75-1.25)	0.79	NA	NA	0.97 (0.78-1.20)	0.76	0.94 (0.77-1.14)	0.51
Q4 (20.5-30)	1.09 (0.86-1.40)	0.47	1.08 (0.82-1.43)	0.57	NA	NA	1.12 (0.88-1.42)	0.34	1.08 (0.88-1.34)	0.45
P trend		0.73		0.88	NA	NA		0.56		0.75

CHD, coronary heart disease; CVD, cardiovascular disease; HR, hazard ratio; NA, not applicable as analyses could not be conducted.

Cox regression model adjusted for age, ethnicity, education, marital status, hysterectomy history, BMI, physical activity, smoking, alcohol intake, region in the U.S, study arm, energy intake, cancer status, hypertension status, diabetes status, sodium intake, family history of CVD, family history of diabetes, hormone therapy use, cholesterol lowering medication use. Under/over energy reporters and those with baseline CVD were excluded from the analysis. [*]No atrial fibrillation cases diagnosed in the first 3 years

Table S5. Associations between high to low adherence to the individual components of the Portfolio diet and risk of cardiovascular outcomes.

Portfolio Diet Component	Total CVD	CHD	Stroke	Heart Failure	Atrial fibrillation
	**HR (95% CI)**	**HR (95% CI)**	**HR (95% CI)**	**HR (95% CI)**	**HR (95% CI)**
Plant protein sources	0.99 (0.93-1.06)	1.00 (0.91-1.11)	1.03 (0.92-1.15)	1.03 (0.87-1.22)	1.02 (0.80-1.30)
P value	0.89	0.90	0.59	0.74	0.88
Nuts	0.90 (0.84-0.95)	0.92 (0.83-1.01)	0.94 (0.84-1.05)	0.78 (0.66-0.92)	0.98 (0.76-1.25)
P value	0.001	0.07	0.29	0.004	0.86
Viscous fiber sources	0.95 (0.89-1.01)	0.92 (0.84-1.01)	1.01 (0.91-1.13)	0.90 (0.77-1.07)	1.00 (0.80-1.26)
P value	0.09	0.08	0.82	0.23	0.99
Plant sterols	0.90 (0.82-0.98)	0.83 (0.73-0.95)	0.84 (0.72-0.98)	0.87 (0.69-1.10)	1.02 (0.73-1.42)
P value	0.02	0.006	0.03	0.24	0.90
MUFA sources	0.92 (0.87-0.97)	0.93 (0.85-1.01)	0.95 (0.86-1.05)	0.90 (0.77-1.04)	1.04 (0.84-1.29)
P value	0.003	0.09	0.30	0.16	0.71
Low saturated fat sources*	0.89 (0.83-0.96)	0.90 (0.80-0.99)	0.95 (0.84-1.07)	0.87 (0.72-1.05)	0.99 (0.76-1.30)
P value	0.002	0.04	0.41	0.14	0.95

CHD, coronary heart disease; CVD, cardiovascular disease; HR, hazard ratio.

The hazard ratios (HRs) are for comparing participants in Q1 (low adherence) to Q5 (high adherence) to the Portfolio Diet components at baseline and year 3 (cumulative average).
The Cox regression model was adjusted for age, ethnicity, education, marital status, hysterectomy history, BMI, physical activity, smoking, alcohol intake, region in the U.S, study arm, energy intake, cancer status, hypertension status, diabetes status, sodium intake, family history of CVD, family history of diabetes, hormone therapy use, cholesterol lowering medication use.
Under/over energy reporters and those with baseline CVD were excluded from the analysis.

*Saturated fat source quintiles were reversed (i.e. Q5 is lowest adherence, Q1 is highest adherence).

Figure S1. Subgroup analyses of the association between low adherence (Q1) to high adherence (Q4) of the Portfolio Diet score and total CVD.

Subgroup	Cases	Total n	HR (95% CIs)	P for interaction
**All participants**	13,365	104,894	0.89 (0.83, 0.94)	
**Age category**				0.09
<50-59	1,878	37,265	0.82 (0.70, 0.96)	
60-69	5,276	47,242	0.96 (0.88, 1.05)	
70+	3,943	20,387	0.88 (0.79, 0.97)	
**BMI category**				0.36
<25 kg/m2	3,198	38,255	0.88 (0.79, 0.99)	
25 to <30 kg/m2	3,929	35,588	0.92 (0.82, 0.99)	
>= 30 kg/m2	3,970	30,051	0.85 (0.76, 0.94)	
**Family history of CVD**				0.07
Yes	8,488	71,899	0.91 (0.84, 0.97)	
No	3,227	38,565	0.79 (0.70, 0.89)	
**Ethnicity**				0.11
White	9,565	88,696	0.87 (0.82, 0.93)	
African American	964	8,175	0.92 (0.73, 1.16)	
Hispanic	273	3,869	0.92 (0.63, 1.36)	
Asian	182	3,018	1.53 (0.94, 2.49)	
**Smoking status**				0.11
Never	5,430	53,820	0.91 (0.83, 0.99)	
Past	4,661	44,015	0.89 (0.81, 0.98)	
Current	1,006	7,059	0.74 (0.58, 0.94)	
**Cholesterol medication use**				0.5
Yes	1,731	12,109	0.85 (0.72, 0.99)	
No	9,366	92,785	0.89 (0.83, 0.96)	

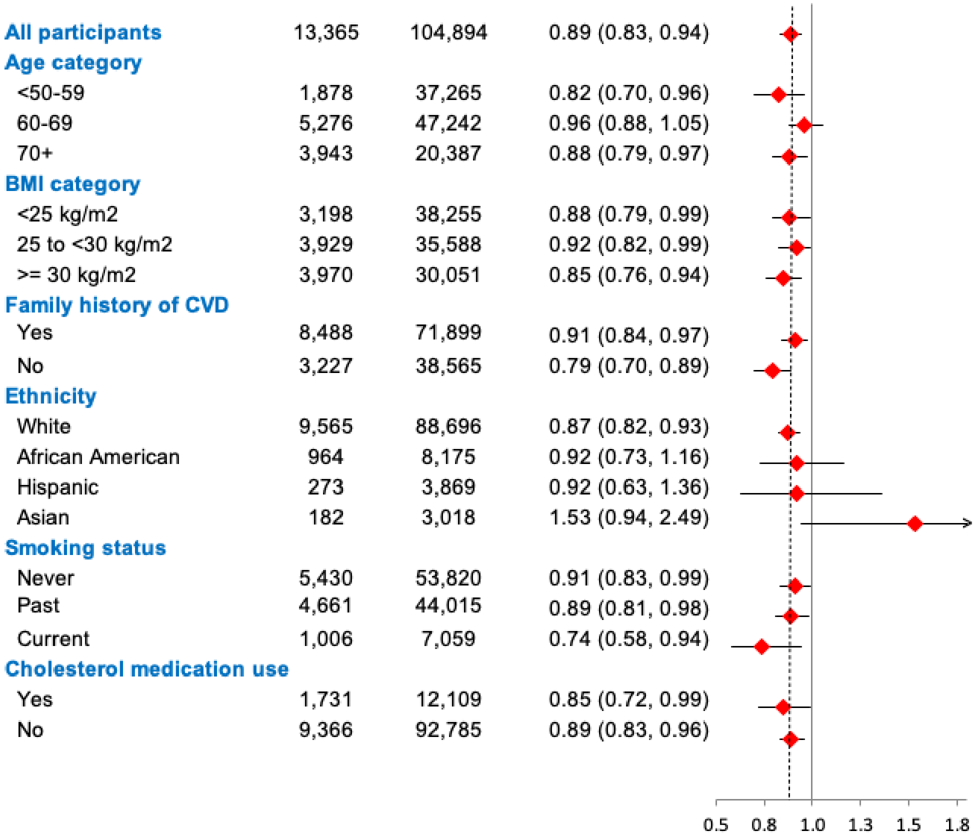

BMI, body mass index; CVD, cardiovascular disease; HR, hazard ratio

The hazard ratios are for comparing participants in Q1 (low adherence) to Q4 (high adherence) to the Portfolio Diet score. The Cox regression model was adjusted for age, ethnicity, education, marital status, hysterectomy history, BMI, physical activity, smoking, alcohol intake, region in the U.S, study arm, energy intake, cancer status, hypertension status, diabetes status, sodium intake, family history of CVD, family history of diabetes, hormone therapy use, and cholesterol lowering medication use. *P* for interaction is comparing participants in Q1 (low adherence) to Q4 (high adherence) to the Portfolio Diet score. Under/over energy reporters and those with baseline CVD were excluded from the analysis.

Figure S2. Subgroup analyses of the association between low adherence (Q1) to high adherence (Q4) of the Portfolio Diet score and CHD.

Subgroup	Cases	Total n	HR (95% CIs)	P for interaction
**All participants**	5,640	104,894	0.86 (0.78-0.95)	
**Age category**				0.05
<50-59	746	37,265	0.77 (0.59, 0.98)	
60-69	2,161	47,243	0.88 (0.76, 1.01)	
70+	1,823	20,389	0.94 (0.81, 1.09)	
**BMI category**				0.26
<25 kg/m2	1,392	38,258	0.84 (0.71, 1.00)	
25 to <30 kg/m2	1,672	36,588	0.84 (0.72, 0.99)	
>= 30 kg/m2	1,666	30,051	0.89 (0.76, 1.05)	
**Family history of CVD**				0.21
Yes	3,658	71,901	0.89 (0.80, 1.00)	
No	1,334	38,566	0.76 (0.64, 0.91)	
**Ethnicity**				0.61
White	4,129	88,699	0.88 (0.79, 0.97)	
African American	383	8,175	0.68 (0.46, 0.98)	
Hispanic	98	3,869	0.81 (0.41, 1.60)	
Asian	73	3,018	1.70 (0.77, 3.76)	
**Smoking status**				0.02
Never	2,278	53,820	0.86 (0.75, 0.99)	
Past	1,953	44,018	0.93 (0.80, 1.08)	
Current	499	7,059	0.60 (0.42, 0.86)	
**Cholesterol medication use**				0.27
Yes	733	12,109	0.75 (0.59, 0.96)	
No	3,997	92,788	0.88 (0.80, 0.98)	

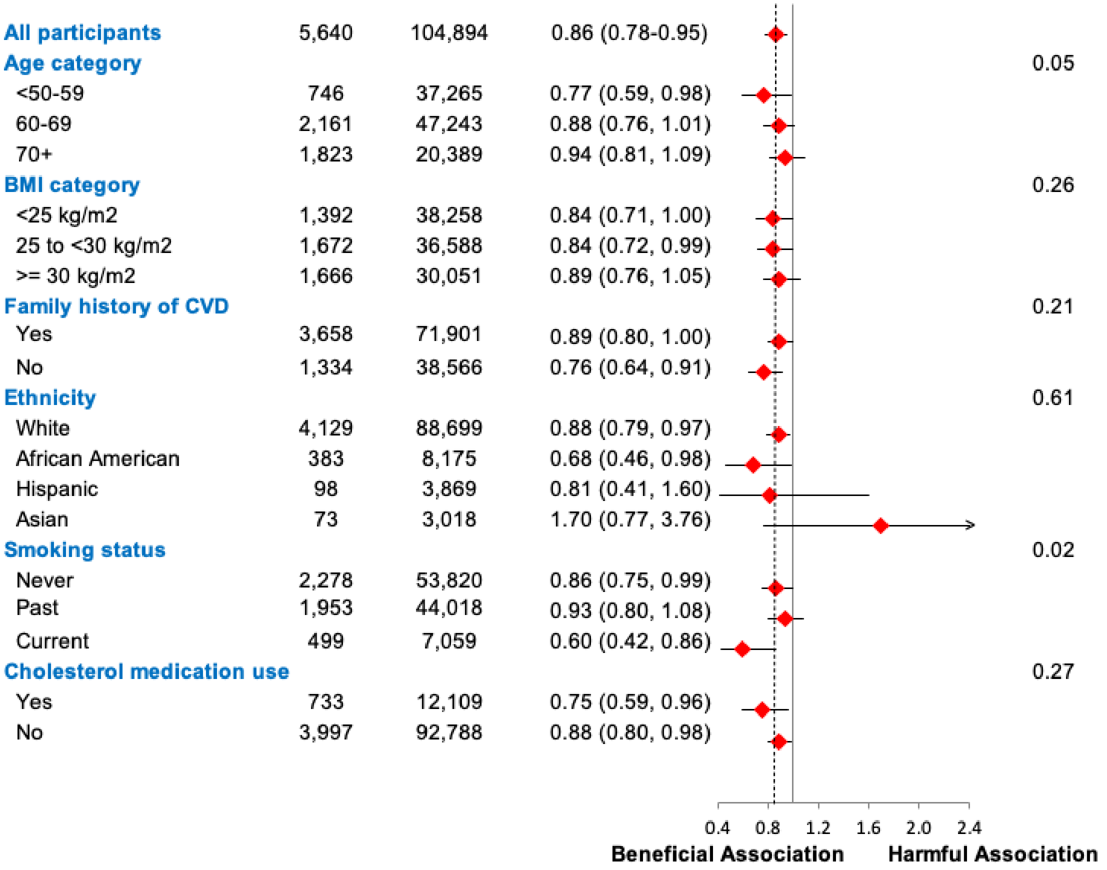

BMI, body mass index; CHD, coronary heart diseases; CVD, cardiovascular disease; HR, hazard ratio

The hazard ratios are for comparing participants in Q1 (low adherence) to Q4 (high adherence) to the Portfolio Diet score. The Cox regression model was adjusted for age, ethnicity, education, marital status, hysterectomy history, BMI, physical activity, smoking, alcohol intake, region in the U.S, study arm, energy intake, cancer status, hypertension status, diabetes status, sodium intake, family history of CVD, family history of diabetes, hormone therapy use, and cholesterol lowering medication use. *P* for interaction is comparing participants in Q1 (low adherence) to Q4 (high adherence) to the Portfolio Diet score. Under/over energy reporters and those with baseline CVD were excluded from the analysis

Figure S3. Subgroup analyses of the association between low adherence (Q1) to high adherence (Q4) of the Portfolio Diet score and Stroke.

Subgroup	Cases	Total n	HR (95% CIs)	P for interaction
All participants	4,440	104,894	0.97 (0.87-1.08)	
Age category				0.38
<50-59	514	37,265	1.09 (0.81, 1.47)	
60-69	1,658	47,242	1.11 (0.94, 1.30)	
70+	1,389	20,389	0.86 (0.72-1.03)	
BMI category				0.65
<25 kg/m2	1,119	38,257	0.98 (0.81, 1.19)	
25 to <30 kg/m2	1,304	36,588	1.07 (0.89, 1.28)	
>= 30 kg/m2	1,138	30,051	0.88 (0.72, 1.07)	
Family history of CVD				0.81
Yes	2,694	71,901	0.96 (0.85, 1.09)	
No	1,096	38,565	0.95 (0.78, 1.15)	
Ethnicity				0.11
White	2,996	88,698	0.93 (0.83, 1.05)	
African American	355	8,175	1.17 (0.81, 1.68)	
Hispanic	90	3,869	1.07 (0.55, 2.09)	
Asian	81	3,018	1.35 (0.68-2.71)	
Smoking status				0.44
Never	1,786	53,820	1.02 (0.87-1.18)	
Past	1,478	44,017	0.94 (0.80, 1.12)	
Current	297	7,059	0.88 (0.57, 1.36)	
Cholesterol medication use				0.18
Yes	487	12,109	1.40 (1.03, 1.88)	
No	3,074	92,787	0.92 (0.81, 1.03)	

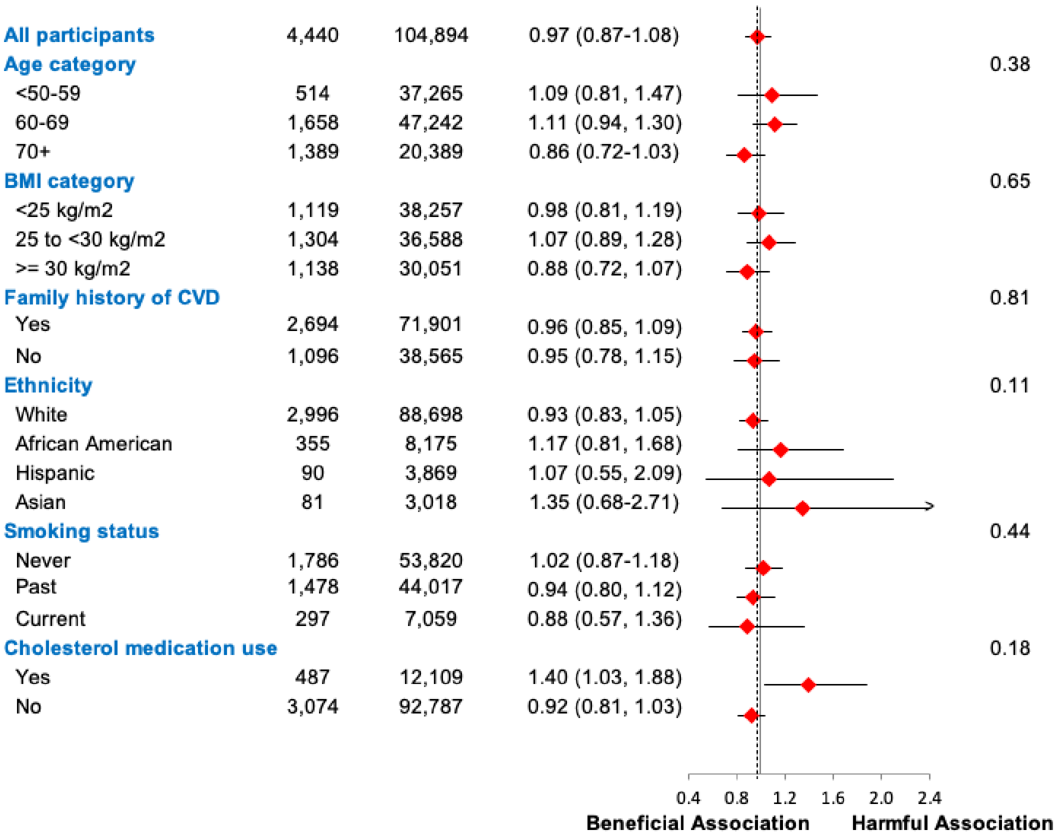

BMI, body mass index; CVD, cardiovascular disease; HR, hazard ratio

The hazard ratios are for comparing participants in Q1 (low adherence) to Q4 (high adherence) to the Portfolio Diet score. The Cox regression model was adjusted for age, ethnicity, education, marital status, hysterectomy history, BMI, physical activity, smoking, alcohol intake, region in the U.S, study arm, energy intake, cancer status, hypertension status, diabetes status, sodium intake, family history of CVD, family history of diabetes, hormone therapy use, and cholesterol lowering medication use. *P* for interaction is comparing participants in Q1 (low adherence) to Q4 (high adherence) to the Portfolio Diet score. Under/over energy reporters and those with baseline CVD were excluded from the analysis.

Figure S4. Subgroup analyses of the association between low adherence (Q1) to high adherence (Q4) of the Portfolio Diet score and Heart Failure.

Subgroup	Cases	Total n	HR (95% CIs)	P for interaction
**All participants**	1,907	104,894	0.83 (0.71, 0.99)	
**Age category**				0.97
<50-59	202	37,265	0.87 (0.54, 1.42)	
60-69	676	47,243	0.96 (0.73, 1.13)	
70+	701	20,387	0.77 (0.60, 0.99)	
**BMI category**				0.12
<25 kg/m2	401	38,256	0.91 (0.66, 1.24)	
25 to <30 kg/m2	444	36,588	1.03 (0.75, 1.43)	
>= 30 kg/m2	734	30,051	0.65 (0.50, 0.84)	
**Family history of CVD**				0.89
Yes	1,183	71,899	0.81 (0.67, 0.98)	
No	491	38,566	0.83 (0.62, 1.12)	
**Ethnicity**				0.99
White	1,371	88,697	0.82 (0.69, 0.99)	
African American	148	8,175	0.93 (0.51, 1.67)	
Hispanic	30	3,869	0.84 (0.28, 2.54)	
Asian	14	3,018	1.41 (0.26, 7.54)	
**Smoking status**				0.81
Never	739	53,820	0.76 (0.59, 0.96)	
Past	686	44,016	0.92 (0.72, 1.17)	
Current	154	7,059	0.83 (0.45, 1.53)	
**Cholesterol medication use**				0.34
Yes	230	12,109	0.58 (0.37, 0.91)	
No	1,349	92,786	0.89 (0.74, 1.06)	

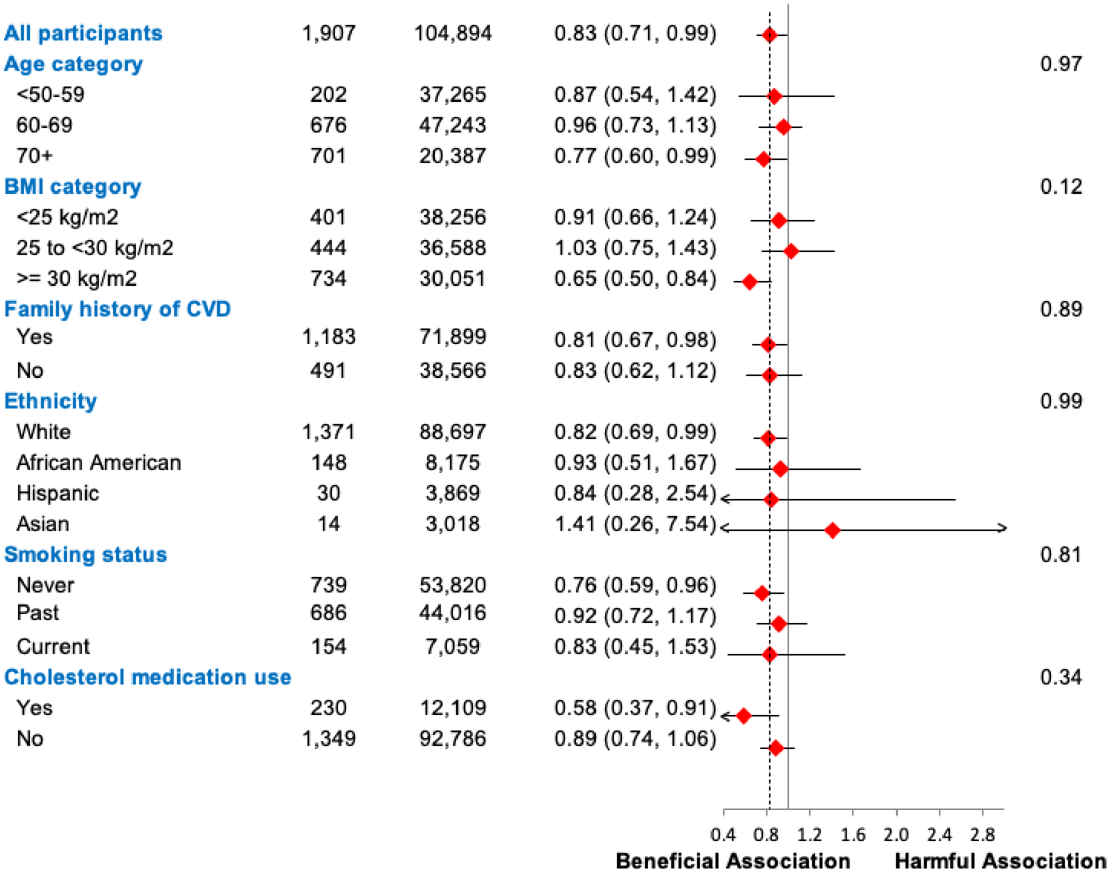

BMI, body mass index; CVD, cardiovascular disease; HR, hazard ratio

The hazard ratios are for comparing participants in Q1 (low adherence) to Q4 (high adherence) to the Portfolio Diet score. The Cox regression model was adjusted for age, ethnicity, education, marital status, hysterectomy history, BMI, physical activity, smoking, alcohol intake, region in the U.S, study arm, energy intake, cancer status, hypertension status, diabetes status, sodium intake, family history of CVD, family history of diabetes, hormone therapy use, and cholesterol lowering medication use. *P* for interaction is comparing participants in Q1 (low adherence) to Q4 (high adherence) to the Portfolio Diet score. Under/over energy reporters and those with baseline CVD were excluded from the analysis.

Figure S5. Subgroup analyses of the association between low adherence (Q1) to high adherence (Q4) of the Portfolio Diet score and Atrial Fibrillation.

Subgroup	Cases	Total n	HR (95% CIs)	P for interaction
**All participants**	929	104,894	1.10 (0.87, 1.38)	
**Age category**				0.41
<50-59	165	37,265	1.47 (0.90, 2.41)	
60-69	416	47,243	0.98 (0.71, 1.35)	
70+	191	20,389	1.30 (0.81, 2.08)	
**BMI category**				0.51
<25 kg/m2	169	38,258	1.18 (0.72, 1.95)	
25 to <30 kg/m2	266	36,588	1.16 (0.78, 1.72)	
>= 30 kg/m2	337	30,051	1.00 (0.70, 1.43)	
**Family history of CVD**				0.43
Yes	560	71,901	0.93 (0.71, 1.23)	
No	251	38,566	1.59 (1.07, 2.37)	
**Ethnicity**				0.18
White	629	88,699	1.06 (0.82, 1.38)	
African American	84	8,175	0.92 (0.44, 1.91)	
Hispanic	51	3,869	4.12 (1.30, 13.04)	
Asian	2	3,018	NA	
**Smoking status**				0.75
Never	354	53,820	0.99 (0.70, 1.39)	
Past	364	44,018	1.20 (0.86, 1.67)	
Current	54	7,059	1.43 (0.53, 3.84)	
**Cholesterol medication use**				0.45
Yes	95	12,109	0.82 (0.41, 1.65)	
No	677	92,788	1.13 (0.88, 1.44)	

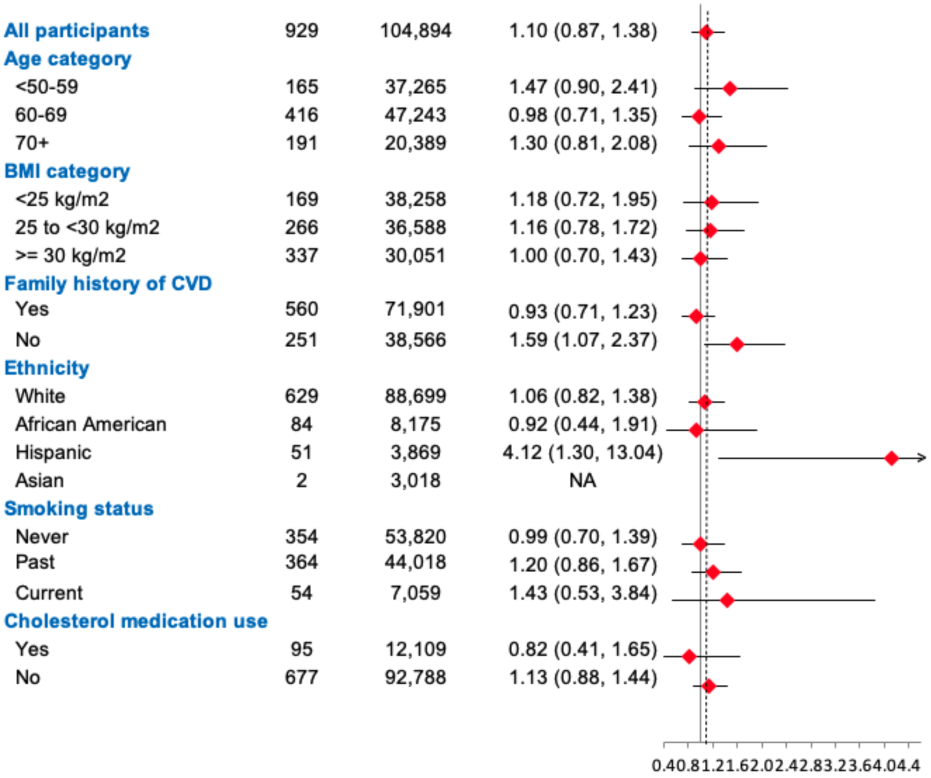

Beneficial Association **Harmful Association**

BMI, body mass index; CVD, cardiovascular disease; HR, hazard ratio; NA, not applicable (too few cases in subgroup).

The hazard ratios are for comparing participants in Q1 (low adherence) to Q4 (high adherence) to the Portfolio Diet score. The Cox regression model was adjusted for age, ethnicity, education, marital status, hysterectomy history, BMI, physical activity, smoking, alcohol intake, region in the U.S, study arm, energy intake, cancer status, hypertension status, diabetes status, sodium intake, family history of CVD, family history of diabetes, hormone therapy use, and cholesterol lowering medication use. *P* for interaction is comparing participants in Q1 (low adherence) to Q4 (high adherence) to the Portfolio Diet score. Under/over energy reporters and those with baseline CVD were excluded from the analysis.
